# Alterations in dopaminergic innervation and receptors in focal cortical dysplasia

**DOI:** 10.1093/brain/awaf080

**Published:** 2025-04-16

**Authors:** Norisa Meli, Katherine Sheran, Julika Pitsch, Sabine Krabbe, Valeri Borger, Tobias Baumgartner, Albert Becker, Sandra Blaess

**Affiliations:** Neurodevelopmental Genetics, Institute of Reconstructive Neurobiology, Medical Faculty, University of Bonn, D-53127 Bonn, Germany; Institute for Cellular Neurosciences II, Medical Faculty, University of Bonn, D-53127 Bonn, Germany; German Center for Neurodegenerative Diseases (DZNE), D-53127 Bonn, Germany; Department of Epileptology, University Hospital Bonn, D-53127 Bonn, Germany; German Center for Neurodegenerative Diseases (DZNE), D-53127 Bonn, Germany; Department of Neurosurgery, University Hospital Bonn, D-53127 Bonn, Germany; Department of Epileptology, University Hospital Bonn, D-53127 Bonn, Germany; Institute for Cellular Neurosciences II, Medical Faculty, University of Bonn, D-53127 Bonn, Germany; Neurodevelopmental Genetics, Institute of Reconstructive Neurobiology, Medical Faculty, University of Bonn, D-53127 Bonn, Germany

**Keywords:** cortical malformations, neurodevelopmental disorders, epileptogenesis, neurotransmitter systems, neuromodulation

## Abstract

Focal cortical dysplasia (FCD) type 2 is the most common malformation of cortical development associated with pharmaco-resistant focal epilepsy and frequently located in the frontal cortex. Neuropathological hallmarks comprise abnormal cortical layering and enlarged, dysmorphic neuronal elements. Fundamentally altered local neuronal activity has been reported in human FCD type 2 epilepsy surgical biopsies. Of note, FCD type 2 emerges during brain development and forms complex connectivity architectures with surrounding neuronal networks. Local cortical microcircuits, particularly in frontal localization, are extensively modulated by monoaminergic axonal projections originating from the brainstem. Previous analysis of monoaminergic modulatory inputs in human FCD type 2 biopsies suggested altered density and distribution of these monoaminergic axons; however, a systematic investigation is still pending.

Here, we perform a comprehensive analysis of dopaminergic (DA) innervation, in human FCD type 2 biopsies and in the medial prefrontal cortex (mPFC) of an FCD type 2 mouse model [mechanistic target of rapamyin (mTOR) hyperactivation model] during adolescent and adult stages. In addition, we analyse the expression of dopamine receptor transcripts via multiplex fluorescent RNA *in situ* hybridization in human specimens and the mPFC of this mouse model.

In the mTOR hyperactivation mouse model, we observe a transient alteration of DA innervation density during adolescence and a trend towards decreased innervation in adulthood. In human FCD type 2 areas, the overall DA innervation density is decreased in adult patients compared with control areas from these patients. Moreover, the DA innervation shows an altered lamination pattern in the FCD type 2 area compared with the control area. Dopamine receptors 1 and 2 appear to be differentially expressed in the dysmorphic neurons in human samples and mTOR-mutant cells in mice compared with normally developed neurons.

Intriguingly, our results suggest complex molecular and structural alterations putatively inducing impaired DA neurotransmission in FCD type 2. We hypothesize that this may have important implications for the development of these malformations and the manifestation of seizures.

## Teaser

Focal cortical dysplasia (FCD) type 2 is one of the most common malformations of cortical development associated with treatment-resistant epilepsy. Using human FCD type 2 biopsies and a mouse model, Meli *et al.* show that affected brain regions exhibit reduced dopaminergic innervation and altered patterns of dopamine receptor expression.

## Introduction

Focal cortical dysplasias (FCDs) comprise a large spectrum of developmental aberrations ranging from abnormal migration (type 1) to fundamentally dysplastic cellular appearance and structure (type 2) of neuronal elements.^1^ FCDs are increasingly recognized as causes of pharmaco-refractory epilepsies.^2^ FCDs, in particular FCD type 2 (subtype ‘a’ lacking so-called balloon cells, and subtype ‘b’ encountering balloon cells), are preferentially localized to the frontal lobe.^1^ Causative gene mutations that result in altered mTOR (mechanistic target of rapamycin) pathway activation have been identified in up to 63% of FCD type 2 spectrum lesions.^3^ However, this increasing knowledge of FCD molecular genetics has not led to tailored antiepileptogenic therapies yet.^4^ This treatment gap may result from a predominantly genetic perspective on the underlying mechanisms. Importantly, dysmorphic FCD neurons exhibit abnormal functional properties and it has been shown in FCD mouse models, that somatic mutations in mTOR or its regulators in only 1%–2% of neurons may be sufficient to cause epileptogenesis.^5-10^ These data strongly suggest that unravelling the pathophysiology of FCDs will require a deeper understanding of how relatively few neurons with altered physiology interact with and affect local circuits and long-range inputs in the developing cortex to lead to the formation of hyperexcitable FCD networks.

The cortex, and especially the frontal lobe, is innervated by several modulatory systems, including dopaminergic (DA), serotonergic (5-HT) and noradrenergic (NA) projections from the brainstem, which influence excitation/inhibition balance of local cortical networks.^11-14^ Of these neuromodulatory systems, DA innervation shows a particularly protracted maturation, which has been explored in detail in the medial prefrontal cortex (mPFC): innervation density increases post-natally from juvenile stages up to early adulthood. Thus, the maturation of the DA system occurs in parallel with, and is also known to influence, the maturation of local cortical circuits in the mPFC in rodents and primates.^15^ In the adult mPFC, dopamine is essential for maintaining the functionality of neural circuits underlying mPFC-mediated cognitive processes and behaviours by regulating neuronal excitability and synaptic plasticity.^16-19^ Accordingly, dysregulation of DA neurotransmission in the PFC contributes to cognitive deficits, emotional dysregulation and impaired executive functions in a number of neuropsychiatric diseases. Alterations of DA modulatory systems are also linked to changes in susceptibility to seizures, but the effect on network activity has primarily been explored in the hippocampus.^8,20,21^ In addition, DA dysregulation may contribute to the manifestation of depressive symptoms frequently observed in patients suffering from FCDs,^22,23^ and compromised DA innervation has been described in human FCD biopsy tissue in a few case studies.^24,25^ Given these functions of dopamine in the developing and mature cortex, altered DA innervation and dopamine-mediated modulation may contribute to hyperexcitability in FCD, but a systematic analysis of potential changes in DA innervation in FCD has not yet been performed.

Dopamine exerts its function through dopamine receptors (DRDs), a class of G protein-coupled proteins that are expressed in both excitatory pyramidal neurons and inhibitory interneurons within the mPFC.^18,26^ DRDs are classified into D1-like (DRD1 and DRD5) or D2-like (DRD2, DRD3 and DRD4) subfamilies, and DRD1 and DRD2 are particularly prominent in the adult mPFC.^27,28^ Similar to DA innervation, DRDs have been reported to be expressed at different levels at distinct post-natal stages and different cortical layers.^19^ Thus, to understand a potential contribution of altered DA modulation on hyperexcitability of local networks in FCD another important aspect is to examine whether DRD expression is altered in the FCD lesion areas.

In the current study, we examined long-range DA modulatory inputs and dopamine receptor transcript expression in FCD lesion areas in human biopsy samples from patients with FCD type 2b and in an animal model of FCD type 2, in which a hyperactivated mTOR is introduced into a subset of cortical progenitors.^9^ To account for potential changes in the maturation of the DA system in FCD, we performed this analysis at adolescent and adult stages. We found that the lamination pattern of DA innervation is altered in FCD lesions in adult, but not in paediatric/adolescent specimens from human FCD type 2b patients. In the mTOR hyperactivation mouse model, we observed a transient change in DA innervation density during adolescence and a trend towards decreased innervation in adulthood. *Drd1/DRD1* and *Drd2/DRD2* receptors are expressed at higher levels in dysmorphic neurons of the FCD area in human patient samples and in mTOR-mutated neurons in mice compared with normal/non-mutated neurons. Taken together, our results demonstrate an altered DA system in FCD lesion areas. We hypothesize that these alterations may lead to aberrant DA neurotransmission in FCD type 2b, potentially contributing to hyperexcitability. Our results pave the way for further investigation into functional changes in DA signalling in FCD type 2b and the potential of targeted stimulation or inhibition of modulatory inputs in FCDs as a means of influencing aberrant network and seizure activity.

## Materials and methods

### Animals

For *in utero* electroporation (IUE) experiments on embryonic Day (E) 14 embryos, timed-pregnant CD1 females were purchased from Charles River (Strain code: #022). Only successfully electroporated offspring were included in the analysis and the number of animals used in each experiment is stated in the figure legends. All experiments were performed according to the welfare animal regulations of the Federal German Government, European Union and the University of Bonn Medical Centre Animal Care Committee. The experimental protocols were approved by the Landesamt für Natur, Umwelt und Verbraucherschutz Nordrhein-Westfalen (Animal Permit Number: 81-02.04.2019.A294). All animals were housed in a controlled environment, with 12-h light/night cycles and *ad libitum* availability of food and water.

### Intraventricular *in utero* electroporation

A plasmid harbouring an mTOR wild-type (WT-mTOR) (pCIG-mTOR-WT-IRES-EGFP) and a mutant mTOR (pCIG-mTOR-Leu2427Pro-IRES-EGFP) construct were generously provided by Jeong Ho Lee from the Graduate School of Medical Science and Engineering, KAIST, Daejeon, Korea.^9^ A plasmid containing a construct for expression of monomeric red fluorescent protein (mRFP) (pAAV-U6-mRFP) was co-electroporated with the WT-mTOR plasmid to distinguish animals with WT-mTOR from animals with mTOR-Leu2427Pro plasmid post-natally.

The IUE protocol was modified from Roben *et al*.^29^ to specifically target the mPFC. Briefly, we injected either the mTOR-Leu2427Pro (2 μg/μl) or WT-mTOR plasmid (2 μg/μl) together with the pAAV-U6-mRFP plasmid (1.5 μg/μl) into the lateral ventricles of the developing mouse brain at E14 via microcapillaries attached to a pressure pulse generator (Picospritzer III, General Valve Corporation). The positive pole of a triple-electrode was placed frontally on the head of the embryos.^30^ Electroporation was achieved by delivering pulses at 30 V with a pulse duration of 20 ms with a CUY21 SC Square Wave Electroporator (Nepa Gene).

### Patients and neuropathology

All patients were suffering from drug-resistant focal epilepsy and underwent presurgical evaluation at the Department of Epileptology, University Hospital Bonn. The localization and extent of the possible epileptogenic zone was determined by seizure semiology, scalp and generally also stereotactic intracranial EEG, brain MRI and when necessary, fluorodeoxyglucose (FDG) PET, and ictal and interictal single-photon emission computer tomography (SPECT).^31^ All patients underwent epilepsy surgery to achieve seizure freedom. All procedures were conducted in accordance with the Declaration of Helsinki. Potential confounding factors such as the effects of gender were minimized by our approach of intra-individual comparisons of data from control versus lesion areas.

### Immunohistochemistry

#### Mouse tissue

Free-floating coronal sections were blocked at room temperature (RT) with 10% normal donkey serum (NDS) in 0.3% Triton X-100 in PBS (PBST) for 1 h and then incubated overnight at 4°C with primary antibodies anti-tyrosine hydroxylase (TH; RRID:AB_390204), anti-green fluorescent protein (GFP, RRID:AB_10013361), anti-phospho-S6 (pS6, Ser240/244) (RRID:AB_10694233) and anti-noradrenaline transporter (NET; RRID:AB_2665806) (Supplementary Table 1). The following day the sections were incubated for 2 h at RT with secondary antibodies conjugated with Alexa 647 (RRID:AB_2536183) or Alexa 488 (RRID:AB_2535794) at 1:500 concentration (Supplementary Table 1). All antibodies were diluted in 3% NDS/0.3% PBST. Hoechst 33258 (1 μg/ml) was used at a dilution of 1:10 000 to counterstain the nuclei.

#### Human tissue

Formalin-fixed and paraffin embedded (FFPE) sections of 4 μm were deparaffinized in Xylol and a graded ethanol series. After a permeabilization step in 0.3% PBST for 10 min, antigen retrieval was performed by incubating sections in 10 mM sodium citrate buffer for ∼10 min at 100°C. Sections were incubated in 10% NDS/PBT blocking solution for 1 h at RT and incubated overnight at 4°C with primary antibodies: anti-TH (RRID:AB_390204), anti-neuronal nuclei (NeuN, RRID:AB_2619988), anti-Calretinin (RRID:AB_10000342), anti-Neurofilament H (clone SMI32) (RRID:AB_2564642) (Supplementary Table 1). NeuN serves as a general neuronal marker, Calretinin is expressed in layer II/III neurons and SMI32 (Neurofilament H)—in addition to its expression in the soma of dysmorphic neurons—is highly expressed in neuronal processes in layers III and V.^32^ Sections were then incubated for 2 h at RT with secondary antibodies conjugated with: biotin (RRID:AB_234059 or RRID:AB_2340397), Alexa 647 (RRID:AB_2340476) or Alexa 488 (RRID:AB_141607) (Supplementary Table 1). For secondary antibodies labelled with biotin, an extra step of Cy3-Streptavidin incubation was performed for 1 h at RT. All antibodies were diluted in 3% NDS/0.1% PBST.

### Multiplex fluorescent *in situ* hybridization

Multiplex fluorescent RNA *in situ* hybridization (FISH) was performed to detect the expression of *DRD* mRNA in fixed-frozen adult mouse brain sections and FFPE human brain sections using the RNAscope® Multiplex Fluorescent Detection Kit v.2 (323110, ACDBio). The protocol was modified from the manufacturer's manual (User Manual: 323100-USM). The following probes from ACDBio were used: *Drd1* (461901-C3) and *Drd2* (406501-C2) for mouse and *DRD1* (524991) and *DRD2* (553991-C2) for human specimens. Hybridized probes were detected with the TSA Vivid™ Fluorophore Kit 650 (Supplementary Table 1). Prior to FISH, immunohistochemistry (IHC) was performed as described earlier. In mouse tissue, an antibody against NeuN (RRID:AB_2532109) was used to visualize the entire population of neurons; antibodies against GFP (RRID:AB_10013361) or RFP (RRID:AB_2336064) to label the electroporated neurons (Supplementary Table 1). In human FCD type 2b tissue, IHC was performed for NeuN to visualize neurons and for Neurofilament H (SMI32) to label dysmorphic neurons.

## Results

### Establishing a mouse model of cortical malformations for the mPFC

**Figure 1:**
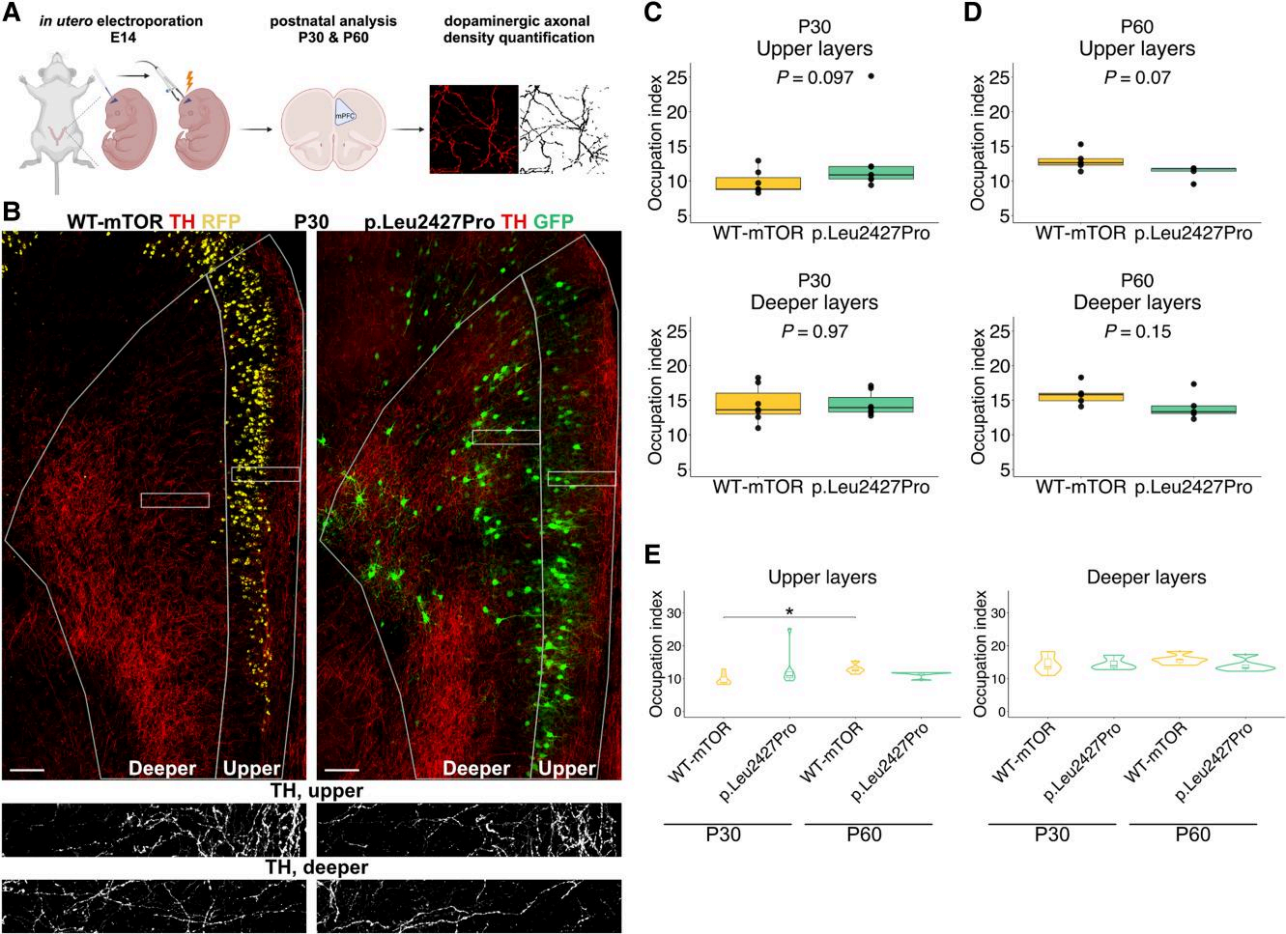
**Altered dopaminergic (DA) innervation density and maturation in the mPFC of the p.Leu2427Pro mTOR mouse model**. (**A**) Experimental pipeline: created in BioRender. Meli, N. (2025) https://BioRender.com/g84c358. (**B**) Representative images of WT-mTOR (*left*) and mTOR p.Leu2427Pro (*right*) mPFC immunostained for TH, GFP and RFP. *Bottom*: Higher magnification of TH+ axons in the boxed regions in the *top* panels. Scale bar = 200 μm. (**C**) Quantification of DA axonal density at P30 (*n =* 7, Mann–Whitney–Wilcoxon test for upper layer and *t*-test for deeper layer). (**D**) Quantification of DA axonal density at P60 (*n* = 5, *t*-test). (**E**) Comparison of DA density quantification between time points in the upper (*left*) and deeper (*right*) layers (Kruskal–Wallis test with *post hoc* Dunn's test with Bonferroni adjustments for multiple comparisons). Occupation index is calculated as the percentage of the area covered by axons in each layer divided by the total area of that layer. The data-points in the box plots represent the mean occupational index for each biological replicate, while the median value is indicated by the horizontal line within the box plot. E = embryonic day; GFP = green fluorescent protein; mPFC = medial prefrontal cortex; mTor = mechanistic target of rapamycin; P = postnatal; RFP = red fluorescent protein; TH = tryosine hydroxylase.

To investigate whether malformations during cortical development affect the density of DA axonal innervation in the mPFC, we first resorted to an mTOR kinase hyperactivation mouse model and adapted it for the mPFC. In this model, introduction of a mutated mTOR (p.Leu2427Pro), first discovered as a somatic mutation in FCD patients, into the developing murine neocortex results in disrupted cortical migration, the presence of cytomegalic neurons and spontaneous seizures.^9^ To target the mTOR mutation to late-born, upper layer pyramidal neurons in the mPFC, we used a modified IUE protocol to deliver a plasmid harbouring the mutated mTOR together with EGFP (p.Leu2427Pro-EGFP) into the progenitors of pyramidal neurons at E14 (Fig. 1A).^30^ As a control, we co-electroporated embryos with a plasmid containing wild-type mTOR plus EGFP (WT-mTOR-EGFP) and a U6-RFP plasmid. RFP expression was used postnatally to distinguish mice with WT-mTOR (GFP+ and RFP+) from mice with mutant mTOR (GFP+). To evaluate the efficiency of this method we examined the distribution of the electroporated cells in the postnatal mPFC. WT-mTOR neurons, which—due to low GFP expression—were identified solely by their RFP expression (RFP+), had migrated to their correct destination in the upper layers. In contrast, neurons electroporated with the mutated mTOR (GFP+) were distributed throughout the cortical layers (Fig. 1B and Supplementary Fig. 1A–D) and showed substantially larger somas than neurons transfected with the WT-mTOR plasmid (Fig. 1B and Supplementary Fig. 1E–L). Mutated mTOR expressing neurons (GFP+) showed a significantly increased expression of pS6 (Ser240/244), a marker for the activated mTOR signalling pathway, compared with surrounding non-electroporated (GFP−) neurons or WT-mTOR expressing neurons (RFP+). In WT-mTOR neurons, the expression of pS6 (Ser240/244) was comparable to the expression in non-electroporated neurons (Supplementary Fig. 2). In summary, targeting p.Leu2427Pro-mTOR into late-born pyramidal neurons in the mPFC recapitulates the phenotypes previously described in more posterior cortical areas.^9^

### Altered maturation of DA innervation density in the mPFC of an mTOR mouse model for cortical malformations

Having successfully established an FCD model in the mouse mPFC, we next analysed the DA axonal density at two postnatal (P) stages, early adolescence (P30) and early adulthood (P60). Mice with IUE of WT-mTOR are referred to as WT-mTOR mice and mice with IUE of p.Leu2427Pro mTOR are referred to as p.Leu2427Pro mice. To label DA axons we performed fluorescent IHC for TH, the rate-limiting enzyme in dopamine synthesis, which is localized at high levels in all cellular compartments of DA neurons, including their axons (Fig. 1B, bottom). Even though TH is also expressed in NA neurons, it is a valid marker to assess the density of DA axons in rodent and human mPFC, since it has been reported that double immunostaining for TH- and NA-neuron specific markers results in only around 10% of NA axons being co-labelled with TH in the human or rat mPFC.^33,34^ Our co-localization analysis of NET (marker for NA axons, Supplementary Fig. 3) and TH in mouse mPFC showed that the overlap is around 26% in the upper layers and around 13% in the deeper layers of the mPFC (Supplementary Fig. 3A and B). Innervation density of TH fibres was measured using a semi-automated analysis pipeline^35^ and expressed as occupation index. Since DA innervation is distinct between the upper and deeper layers of the murine mPFC,^36^ we analysed upper and deeper layers separately (see the Supplementary material, ‘Material and Methods’ section for details on how the layers were defined, Supplementary Fig. 4A–C). At P30, in both the upper and deeper layers of the mPFC, there was no significant difference in the density of DA innervation between the WT-mTOR and p.Leu2427Pro mPFC (Fig. 1C). However, at P60, we observed a trend towards decreased DA innervation in the upper layers of p.Leu2427Pro compared with WT-mTOR mPFC (Fig. 1D). Comparison of DA innervation density between P30 and P60 in p.Leu2427Pro or WT-mTOR mice showed that DA innervation density significantly increased from P30 to P60 in the upper layers of WT-mTOR mPFC, consistent with the known maturation of DA innervation^37^ (Fig. 1E), whereas no significant increase could be detected between the two time points in the p.Leu2427Pro mPFC. Finally, because maturation of DA innervation has been described to differ between mPFC subregions (anterior cingulate, prelimbic and infralimbic cortex in rodents),^37^ we further refined our analysis by examining the density of TH-expressing axons in each mPFC subregion separately (Supplementary Fig. 4D–F). This analysis showed a significant increase of DA axon density in the deeper layers of the P30 anterior cingulate cortex in p.Leu2427Pro mice compared with WT-mTOR mice (Supplementary Fig. 4E). At P60, a significant decrease in innervation density was observed in both upper and deeper layers of the infralimbic cortex in p.Leu2427Pro compared with WT-mTOR mice (Supplementary Fig. 4F). Given that we observed some overlap of NET with TH fibres in the mouse mPFC (Supplementary Fig. 3A and B), we also analysed the density of NET-expressing fibres in the upper and deeper layers of each mPFC subregion in p.Leu2427Pro and in WT-mTOR mice, but did not find any significant differences between the two animal groups (Supplementary Fig. 3C). Taken together, these results show that the mPFC malformations induced in the p.Leu2427Pro mTOR mouse model leads to altered DA maturation and innervation patterns in the mPFC.

### Dopamine receptor expression is upregulated in p.Leu2427Pro mTOR neurons in the mouse mPFC

**Figure 2:**
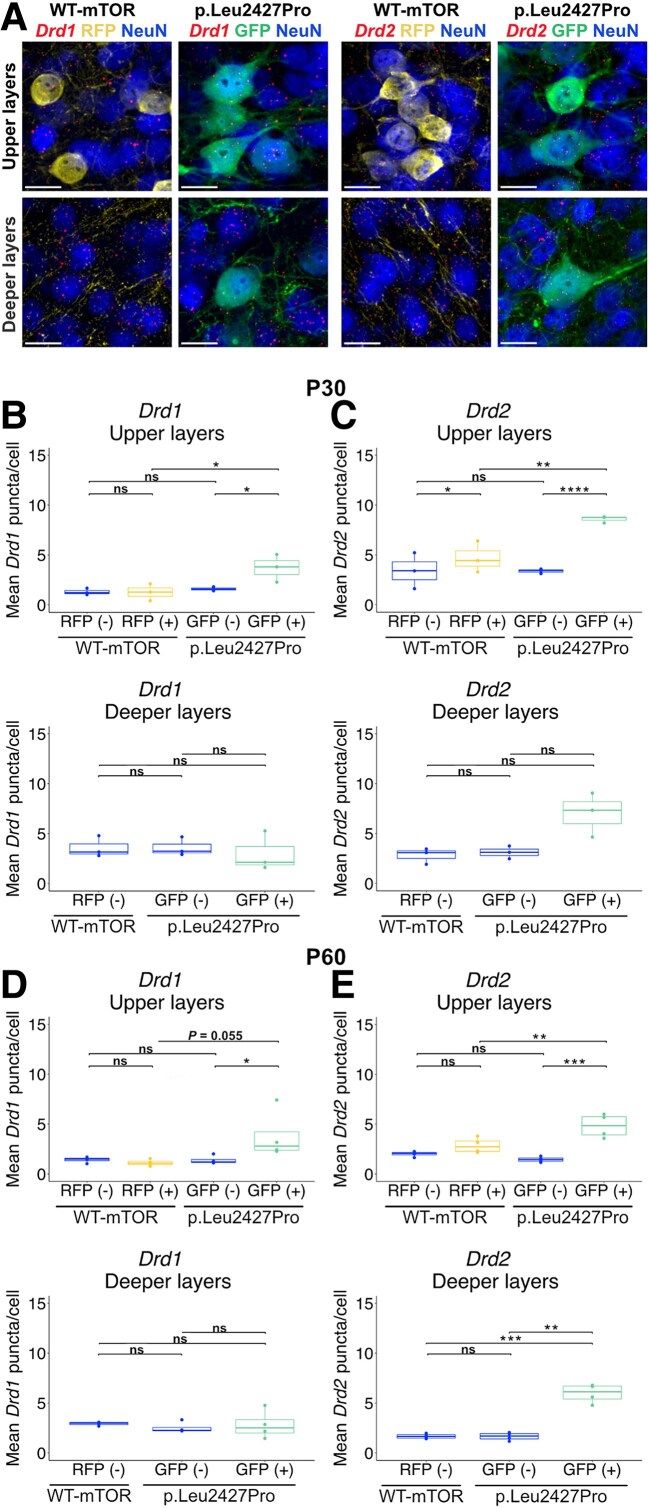
***Drd1* and *Drd2* expression is upregulated in neurons with the p.Leu2427Pro mTOR mutation**. (**A**) Representative images of FISH for *Drd1* (*left*) and *Drd2* (*right*) in combination with NeuN and GFP or RFP immunostaining in the upper and deeper layers of WT-mTOR and p.Leu2427Pro mPFC at P30. Scale bar = 20 μm. (**B**–**E**) Quantification of *Drd1* (**B** and **D**) and *Drd2* (**C** and **E**) mRNA expression levels in different groups of neurons in P30 (**B** and **C**) and P60 (**D** and **E**) WT and p.Leu2427Pro mPFC. Groups in WT-mTOR mPFC: RFP (−): non-electroporated, NeuN+ neurons; RFP (+): neurons electroporated with WT-mTOR plasmid, NeuN+. Groups in p.Leu2427Pro mPFC: GFP (−): non-electroporated, NeuN+ neurons; GFP (+): neurons electroporated with mTOR-pLeu2427Pro plasmid, NeuN+. Number of neurons quantified for *Drd1* analysis at P30: upper layers: 508 RFP (−), 125 RFP (+), 578 GFP (−), 60 GFP (+), deeper layers: 1069 RFP (−), 571 GFP (−), 83 GFP (+). Number of neurons quantified for *Drd2* analysis at P30: upper layers: 572 RFP (−), 124 RFP (+), 527 GFP (−), 69 GFP (+), deeper layers: 1073 RFP (−), 607 GFP (−), 66 GFP (+). Number of neurons quantified for *Drd1* analysis at P60: upper layers: 1123 RFP (−), 88 RFP (+), 1081 GFP (−), 53 GFP (+), deeper layers: 1912 RFP (−), 1231 GFP (−), 60 GFP (+). Number of neurons quantified for *Drd2* analysis at P60: upper layers: 1303 RFP (−), 98 RFP (+), 1069 GFP (−), 72 GFP (+), deeper layers: 1805 RFP (−), 971 GFP (−), 72 GFP (+). P30: *n* = 3 mice per group, P60: *n* = 4 mice per group. Upper layers: two-way ANOVA with repeated measures to compare within experimental groups followed by Bonferroni test to correct for multiple comparisons between the indicated groups. Deeper layers: paired *t*-test to compare GFP (−) versus GFP (+) groups within the p.Leu2427Pro experimental group and unpaired *t*-test to compare between other groups. *P*-values were adjusted for multiple comparisons using the Bonferroni–Dunn method. The data points in the boxplots represent the mean puncta/cell expression for each biological replicate, while the median value is indicated by the horizontal line within the boxplot. *Drd1* = dopamine receptor D1; *Drd2* = dopamine receptor D2; FISH = multiplex fluorescent *in situ* hybridization; GFP = green fluorescent protein; mPFC = medial prefrontal cortex; mTOR = mechanistic target of rapamycin; NeuN = neuronal nuclei marker; P = postnatal; RFP = red fluorescent protein.

The altered density of DA innervation in the mTOR cortical malformation mouse model may ultimately lead to changes in dopamine release and dopamine availability in the mPFC. On the side of the dopamine-receiving neurons, another key factor that determines the efficacy of dopamine neurotransmission and its downstream effect is the expression of the type and level of dopamine receptors (see also Introduction).^18^ To investigate whether the introduction of p.Leu2427Pro mTOR affects dopamine receptor expression in the targeted region in a cell-autonomous or non-cell-autonomous manner, we performed quantitative FISH (see Supplementary material, ‘Material and Methods’ section for details on quantification) for *Drd1* or *Drd2* mRNA in P30 and P60 WT-mTOR or p.Leu2427Pro mPFC in combination with immunostaining for RFP or GFP, respectively, and the general neuronal marker NeuN (also known as RBFOX3) (Fig. 2A). We analysed expression levels in the upper and deeper layers separately, because *Drd1* and *Drd2* have been reported to be predominantly expressed in the deeper layers of rodent mPFC.^26^ Analysis of the WT-mTOR mPFC confirmed that *Drd1* expression was higher in deeper layers [mean ± standard error of the mean (SEM) P30: 3.58 ± 0.615 puncta per cell, P60: 2.93 ± 0.091] than in upper layers (P30: 1.30 ± 0.233, P60: 1.40 ± 0.140). In contrast, we found *Drd2* expression to be slightly higher in the upper layers (P30: 3.65 ± 1, P60: 2.06 ± 0.106) than in the deeper layers (P30: 2.81 ± 0.463, P60: 1.67 ± 0.125) in our samples. In p.Leu2427Pro mPFC, p.Leu2427Pro mTOR expressing neurons (GFP+) in the upper layers expressed significantly more *Drd1* mRNA (P30: 3.71 ± 0.804, P60: 3.60 ± 0.432) than the surrounding non-electroporated neurons (GFP−) (P30: 1.60 ± 0.116, P60: 1.44 ± 0.062) and than RFP+ neurons (P30: 1.27 ± 0.490, P60: 1.17 ± 0.148) in the upper layers of the WT-mTOR mPFC (Fig. 2B and D and Supplementary Figs 5A and 6A) indicating a cell-autonomous effect of the mutation on *Drd1* expression. GFP+ neurons (P30: 3 ± 1.145, P60: 2.81 ± 0.710) in the deeper layers did not show any significant differences in *Drd1* mRNA expression compared with GFP− (P30: 3.60 ± 0.543, P60: 2.51 ± 0.270) neurons in the p.Leu2427Pro mPFC or neurons in the WT-mTOR cortex (P30: 3.57 ± 0.614, P60: 2.93 ± 0.091) (Fig. 2B and D and Supplementary Figs 5A and 6A). Since the GFP+ neurons in the deeper layers are neurons destined for the upper layers, and upper layer neurons normally have relatively low *Drd1* expression, *Drd1* expression levels similar to those of WT-mTOR deeper layer neurons may actually indicate an upregulation of *Drd1* in the p.Leu2427Pro neurons ectopically located in the deeper layers. Indeed, there was no significant difference in *Drd1* expression between upper (P30: 3.71 ± 0.804, P60: 3.60 ± 0.432) and deeper (P30: 3 ± 1.145, P60: 2.81 ± 0.710) layer GFP+ neurons in the p.Leu2427Pro mPFC (Supplementary Figs 5B and 6B) further supporting a cell-autonomous effect of the mutation that is independent of the local conditions.

Analysis of *Drd2* mRNA expression showed that GFP+ cells (P30: 8.59 ± 0.197 puncta per cell, P60: 4.82 ± 0.596) in the upper layers of p.Leu2427Pro mice expressed significantly more *Drd2* mRNA than non-electroporated neurons (GFP−) (P30: 3.37 ± 0.135, P60: 1.45 ± 0.136) or than RFP+ neurons in WT-mTOR mice (P30: 4.70 ± 0.910, P60: 2.86 ± 0.382) mPFC (Fig. 2C and E and Supplementary Figs 5C and 6C). The significant difference in *Drd2* expression between electroporated WT-mTOR (RFP+) and p.Leu2427Pro mTOR expressing (GFP+) neurons indicates a clear cell-autonomous effect of the mutation on *Drd2* expression. Finally, at P60, a significant increase in *Drd2* expression was also detected in the GFP+ cells (P30: 7.01 ± 1.284, P60: 5.96 ± 0.475) in the deeper layers of the mPFC compared with deeper layer GFP− neurons (P30: 3.11 ± 0.370, P60: 1.65 ± 0.20) in the p.Leu2427Pro mice or RFP− neurons (P30: 2.81 ± 0.462, P60: 1.67 ± 0.125) in the WT-mTOR mice (Fig. 2C and E and Supplementary Figs 5C and 6C). There was no significant difference in *Drd2* expression between upper (P30: 8.59 ± 0.197, P60: 4.82 ± 0.596) and deeper (P30: 7.01 ± 1.284, P60: 5.96 ± 0.475) layer GFP+ neurons in the p.Leu2427Pro mPFC (Supplementary Figs 5D and 6D). Finally, to examine whether the increased expression of *Drd1* and *Drd2* in GFP+ neurons might be associated with the greater soma size of these cytomegalic neurons, we performed a correlation analysis between the neuronal volume and *Drd1/Drd2* expression for each neuron group (Supplementary Figs 5E, F, 6E and F). Spearman correlation coefficients did not reveal a strong correlation between cell size and *Drd1* (Supplementary Figs 5E and 6E) or *Drd2* (Supplementary Figs 5F and 6F) transcript expression. In summary, these data show that the expression of mTOR p.Leu2427Pro in neurons destined for the upper cortical layers in the mPFC elicits a strong, cell-autonomous upregulation of *Drd1* and *Drd2* expression that is not obviously due to the increased soma size of the mTOR p.Leu2427Pro-expressing neurons or influenced by their position within the cortical layers.

### Characterization of human FCD type 2b specimens for subsequent DA innervation and receptor analysis

**Figure 3:**
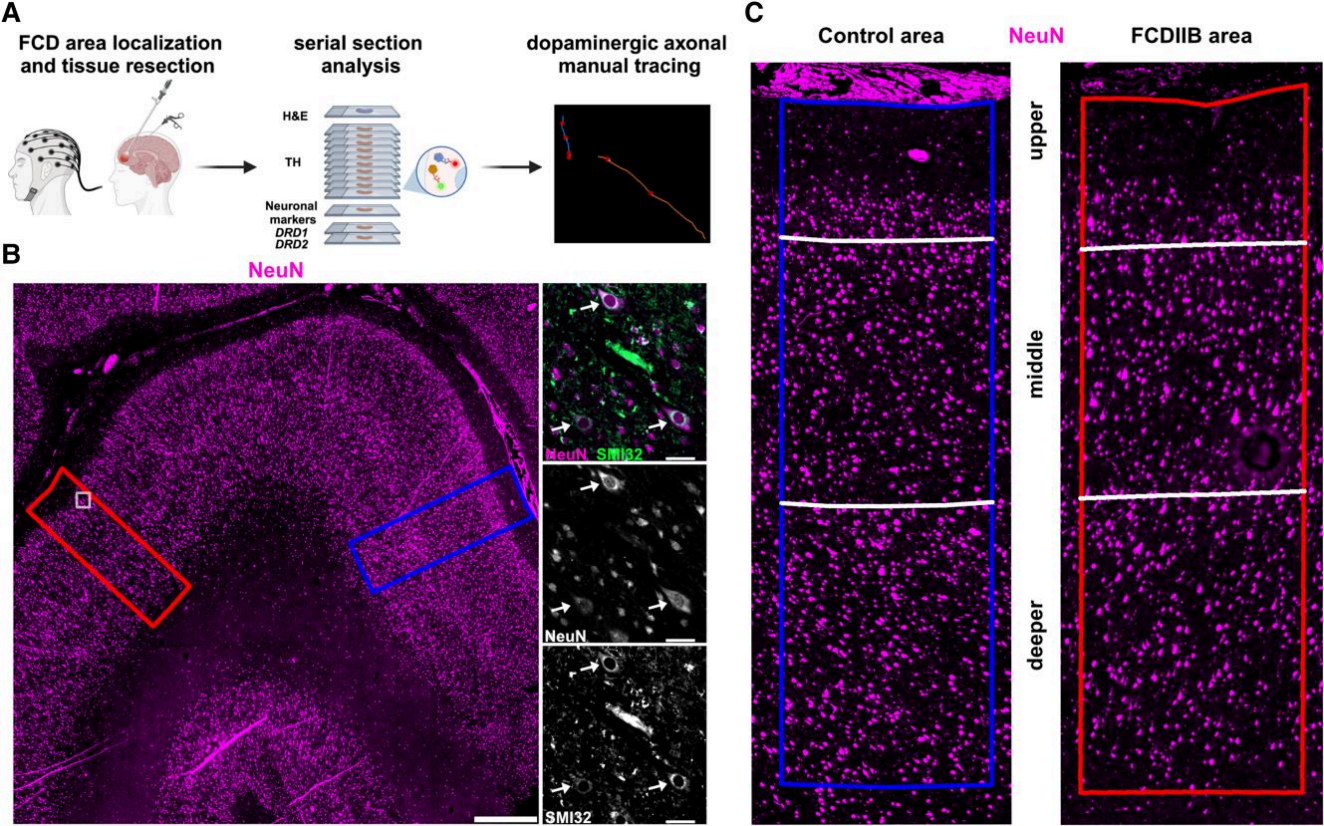
**Characterization of control and FCD type 2b areas in human specimens of FCD type 2b patients**. (**A**) Experimental pipeline: created in BioRender. Meli, N. (2025) https://BioRender.com/z47q234. (**B**) Identification of control and FCD type 2b (FCDIIB) areas within the biopsied tissue. Example images for one patient biopsy. *Left*: Control (blue box, *right*) and FCDIIB (red box, *left*) are defined based on immunostaining for NeuN. Scale bar = 1000 μm. *Right*: Higher magnification of a region in the FCD type 2b area (indicated by the white box in the *left* panel) immunostained for SMI32 and NeuN. SMI32 is expressed in dysmorphic neurons (white arrows). Scale bar = 50 μm. (**C**) Higher magnification of control and FCD type 2b areas indicated with the blue and red boxes from the *left* panel in **B**. Division in upper, middle and deeper layers is indicated. FCD = focal cortical dysplasia; NeuN = neuronal nuclei marker; SMI32 = neurofilament H (antibody, clone SMI32).

**Figure 4:**
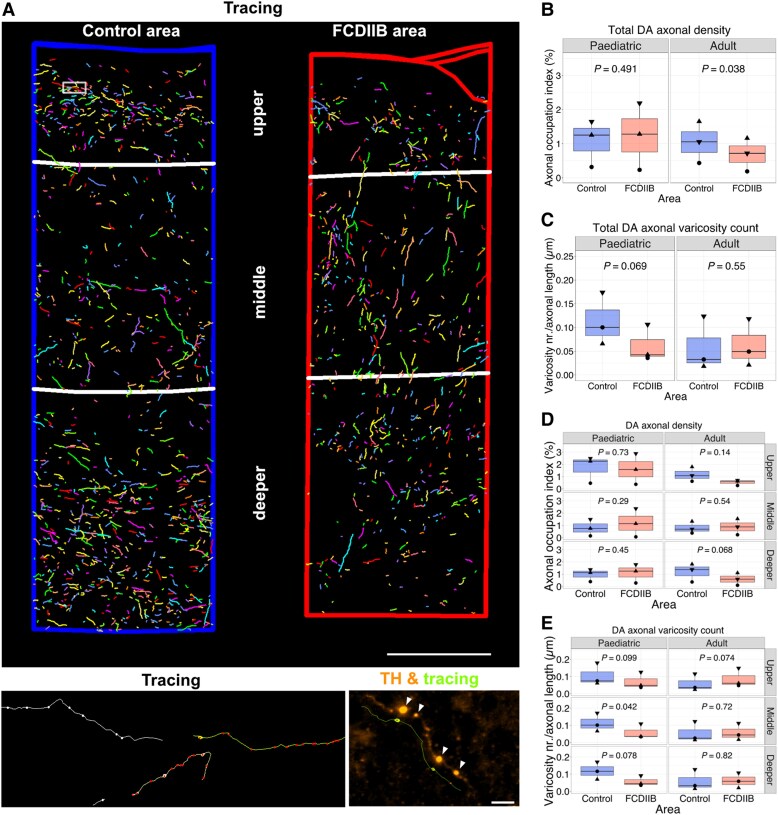
**Altered laminar distribution of dopaminergic (DA) axons and changes in the density of axonal varicosities in the FCD type 2b areas of human specimens**. (**A**) *Top*: 3D reconstruction of DA axonal tracings in control and FCD type 2b (FCDIIB) areas across the upper, middle and deeper layers. Reconstruction of a total of seven serial sections from one adult patient (frontal cortex). Scale bar = 500 μm. *Bottom left*: Higher magnification of boxed area in control region of the *top* panel showing example traces of TH axons in serial sections. Yellow and green axons were traced in one section, while the white axon was traced in an adjacent section. *Bottom right*: Example of varicosities (arrowheads) detected in one TH axon. Scale bar = 10 μm. (**B** and **C**) Quantification of DA axonal density (**B**) and varicosity density along the axons (**C**) in the entire control and FCD type 2b area. (**D** and **E**) Separate quantification of DA axonal density (**D**) and varicosity density along the axons (**E**) in upper, middle and deeper layers in control and FCD type 2b areas from paediatric and adult patients. Occupation index is calculated as percentage of total axonal length for each analysed area normalized for the total area. *n* = 3 specimen for paediatric and for adult patients, paired *t*-test. The data signs in the box plots represent the mean value for each biological replicate, while the median value is indicated by the horizontal line within the box plot. Symbols: filled downward triangle, filled upward triangle = frontal cortex; filled circle = parietal cortex. FCD = focal cortical dysplasia; TH = tyrosine hydroxylase.

Given the changes in DA innervation and *Drd* expression in the mTOR cortical malformation mouse model, we next wanted to investigate if DA innervation and receptor expression is altered in patients with FCD type 2b (Fig. 3A). The age-related maturation of DA innervation described in rodents has also been reported in humans.^19^ Accordingly, the patients analysed were divided into two groups: paediatric (9–16 years of age) and adult (44–57 years of age) (Table 1). These two age groups are comparable to the stages chosen for the analysis of our mouse model. Both frontal and parietal cortex regions were included since—in contrast to rodents—both cortical areas have substantial DA innervation.^33^ In all analyses, FCD type 2b areas were compared with control areas in tissue from the same patient (Fig. 3B). Control areas were defined as areas with proper cortical lamination and no cytological abnormalities. FCD type 2b areas were defined as regions with clear features of cortical dyslamination, presence of dysmorphic neurons with enlarged soma expressing SMI32 (Fig. 3B, white arrows) and balloon cells (not shown). In the human prefrontal and parietal areas, DA innervation exhibits a bi-lamination pattern where the upper and deeper layers are more highly innervated than the middle layers (Fig. 4A).^33^ Thus, we divided the selected cortical regions in upper, middle and deeper layers in the selected control areas in each patient based on the expression of the cortical layer markers (Fig. 3C and Supplementary Fig. 7A). This subdivision into layers was then adapted to define ‘layers’ in the disorganized FCD type 2b areas, taking care that the ratio of layer thickness was similar to the control (Fig. 3C and Supplementary Fig. 7B). The division into layers was applied for the analysis of DA innervation and *DRD1*/*2* expression.

**Table 1:** Human FCD type 2b specimens

Patient no.	Age group	Age at surgical resection, years	Age at seizure onset, years	Localization of the FCD	Gender	Total serial sections	Mean area (µm^2^) control	Mean area (µm^2^) FCD
1	Paediatric	9	8	Frontal	Female	6	7169040	7733350
2	Paediatric	16	3	Frontal	Female	9	2466100	2752870
3	Paediatric	14	1	Parietal	Female	9	7252430	12303500
4	Adult	57	4	Frontal	Male	10	6532400	8983620
5	Adult	45	6	Frontal	Female	7	2401390	2302600
6	Adult	44	5	Parietal	Male	9	29281900	21231200

FCD = focal cortical dysplasia.

### Laminar distribution and density of DA innervation is altered in FCD type 2b areas in human specimens

To analyse the distribution of DA axons in control and FCD type 2b areas (Fig. 3A), we manually traced TH axons in stacks of serial sections using Neurolucida (MBF Bioscience) (Figs 3A and 4A). We also monitored the distribution of axonal varicosities, which—in contrast to the mouse cortex—were easily detectable in the human specimen, since they could represent potential DA release sites (Fig. 4A, bottom right).^38^ The density of the traced axons and the number of varicosities normalized for axon length were then quantified in the fully reconstructed stack of serial sections in FCD type 2b and control areas (Fig. 4A–E). In adult, but not in paediatric specimens, overall DA innervation density was significantly decreased in FCD type 2b areas compared with control regions (Fig. 4B). On the other hand, there was a trend for an overall decreased number of axonal varicosities in FCD type 2b areas compared with control regions in specimens from paediatric but not adult patients (Fig. 4C). To account for the distinct density of DA innervation in different layers of the cortex,^33^ we then analysed DA axon density separately in the upper, middle and deeper layers (Fig. 4A). No apparent difference in innervation density between control and FCD type 2b areas was observed for any of the layers in paediatric patient samples. In contrast, a trend towards decreased DA innervation in the upper and deeper layers in the FCD type 2b area compared with the control area was found in adult patient tissue, therefore disrupting the bi-lamination pattern observed in the control areas as described^33^ (Fig. 4D). In specimens from paediatric, but not from adult patients, analysis of the number of axonal varicosities showed a trend towards a decreased number of varicosities in upper and deeper layers and a significant decrease in the middle layer of the FCD type 2b areas compared with control regions consistent with the analysis of the whole area (Fig. 4E). In conclusion, these results show that DA innervation is impaired in FCD type 2b areas in adult patients, whereas it is the density of varicosities along DA axons that is altered in FCD type 2b areas in paediatric patients.

### Dopamine receptor expression is increased in dysmorphic neurons in FCD type 2b areas in the human cortex

**Figure 5:**
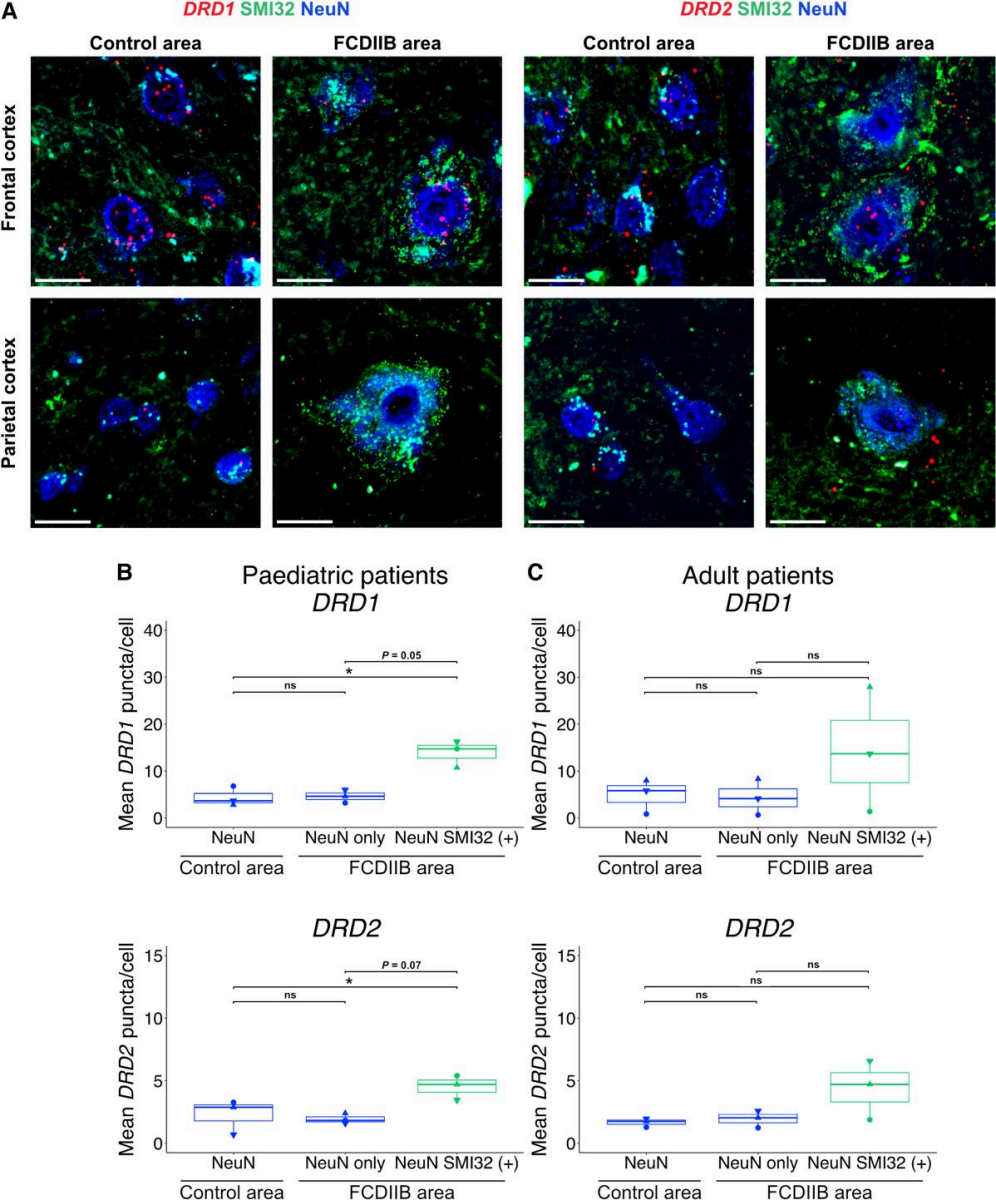
***DRD1* and *DRD2* expression is upregulated in dysmorphic neurons in FCD type 2b cortical areas of paediatric and adult patients**. (**A**) Representative images of FISH for *DRD1* (*left*) and *DRD2* (*right*) in control and FCD type 2b (FCDIIB) areas of adult frontal and parietal cortex. Scale bar = 20 μm. (**B**) Expression analysis of *DRD1* and *DRD2* mRNA in paediatric human FCD specimen. Quantified cells for *DRD1*: 105 NeuN, 39 NeuN only, 26 NeuN SMI32 (+). *DRD2*: 110 NeuN, 37 NeuN only, 31 NeuN SMI32 (+). (**C**) Expression analysis of *DRD1* and *DRD2* mRNA in adult human FCD type 2b specimen. Quantified cells for *DRD1*: 213 NeuN, 101 NeuN only, 42 NeuN SMI32 (+). *DRD2*: 289 NeuN, 100 NeuN only, 38 NeuN SMI32 (+). *n* = 3 specimen for paediatric and for adult patients, one-way ANOVA with repeated measures followed by Tukey's test for multiple comparisons between groups. The data signs in the box plots represent the mean puncta/cell expression for each biological replicate, while the median value is indicated by the horizontal line within the box plot. Symbols: filled downward triangle, filled upward triangle = frontal cortex; filled circle = parietal cortex. *DRD1* = dopamine receptor D1; *DRD2* = dopamine receptor D2; FCD = focal cortical dysplasia; FISH = multiplex fluorescent *in situ* hybridization; NeuN = neuronal nuclei marker; SMI32 = neurofilament H (antibody clone SMI32).

Since we observed changes in the expression of *Drd1* and *Drd2* mRNA in the mTOR-mutant neurons in the cortical malformation mouse model, we next analysed whether *DRD1* or *DRD2* expression levels were altered in FCD type 2b compared with control areas of human specimens. We again performed quantitative FISH for *DRD1* and *DRD2* mRNA in combination with immunostaining for NeuN to label neurons globally and for SMI32 to identify dysmorphic neurons (designated as NeuN SMI32+) (Fig. 5A). Non-dysmorphic, SMI32-negative neurons in the FCD type 2b area were designated as ‘NeuN only’, and neurons in the control area were designated as ‘NeuN’. Quantification of *DRD1* transcripts over all the layers showed that the expression of *DRD1* was upregulated in dysmorphic neurons (13.89 ± 1.651 puncta per cell) compared with NeuN (4.41 ± 1.213) or NeuN-only (4.61 ± 0.804) neurons in paediatric patients. Similarly, *DRD2* was upregulated in dysmorphic neurons (4.51 ± 0.568) compared with NeuN (2.28 ± 0.8) or NeuN-only (1.94 ± 0.237) neurons in paediatric patients (Fig. 5B). In contrast, in adult patients, *DRD1* and *DRD2* transcripts appeared to be only upregulated in dysmorphic neurons in the frontal but not the parietal cortex (Fig. 5C). When cells were pooled across specimens, the dysmorphic cells *DRD1* and *DRD2* appeared to be upregulated in the FCD type 2b areas of adult patients compared with control neurons: dysmorphic neurons expressed higher levels of *DRD1* (13.2 ± 2.04) than NeuN (4.65 ± 0.358) or NeuN-only (4.41 ± 0.672) neurons and higher levels of *DRD2* transcript (3.79 ± 0.646) than NeuN (1.6 ± 0.087) or NeuN-only (1.8 ± 0.207) neurons (Supplementary Fig. 8A and B). Since *DRD1* and *DRD2* have been reported to be expressed at distinct levels in different cortical layers in humans,^19^ we also attempted to analyse *DRD1* and *DRD2* expression separately for different layers (Tables 2 and 3). Since only few dysmorphic neurons were present in the upper layers it was difficult to draw valid conclusions for all the layers (Tables 2 and 3 and Supplementary Fig. 8C and D), but in general, we observed a higher *DRD* expression in dysmorphic neurons in the middle and deeper layers of the FCD type 2b area than in non-dysmorphic neurons in the FCD type 2b or control area. Given that dysmorphic neurons have enlarged cell somas, we next examined if the increased *DRD1* and *DRD2* mRNA expression correlates with soma size. To investigate this, we performed a correlation analysis between the soma size defined by the NeuN immunostaining and the number of *DRD1/DRD2* mRNA puncta per cell. This analysis did not reveal any potential correlation between size and quantity (Supplementary Fig. 9). In conclusion, we demonstrate that expression of *DRDs* is increased in dysmorphic neurons in paediatric and adult FCD type 2b patients. This increase is independent of the increased soma size of the dysmorphic neurons. In addition, we demonstrate that the increase is specific to the dysmorphic neurons, as there is no significant difference in *DRD1* or *DRD2* expression between NeuN neurons in control areas and NeuN-only neurons in the FCD type 2b area.

**Table 2:** *DRD1* expression across layers in pooled cells in human FCD type 2b specimens

Age group	Area	Cell type	Layer	Mean puncta per cell	± SEM
Paediatric	Control	NeuN	Upper	4.45	0.627
			Middle	2.62	0.507
			Deeper	5.59	0.941
	FCDIIB	NeuN only	Upper	6.62	1.76
			Middle	4.17	1.05
			Deeper	3.32	0.761
	FCDIIB	SMI32 (+)	Upper	13.5	5.50
			Middle	15.2	2.13
			Deeper	12.9	1.89
Adult	Control	NeuN	Upper	4.07	0.485
			Middle	4.68	0.732
			Deeper	5.29	0.678
	FCDIIB	NeuN only	Upper	3.13	0.935
			Middle	3.08	0.779
			Deeper	6.97	1.54
	FCDIIB	SMI32 (+)	Upper	8.69	3.57
			Middle	15.4	2.81
			Deeper	14.8	5.02

FCD = focal cortical dysplasia; SEM = standard error of the mean.

**Table 3:** *DRD2* expression across layers in pooled cells in human FCD type 2b specimens

Age group	Area	Cell type	Layer	Mean puncta per cell	± SEM
Paediatric	Control	NeuN	Upper	2.1	0.328
			Middle	2.5	0.429
			Deeper	3.33	0.463
	FCDIIB	NeuN only	Upper	1.5	0.535
			Middle	2.23	0.469
			Deeper	2.31	0.631
	FCDIIB	SMI32 (+)	Upper	3.25	0.854
			Middle	5.08	0.796
			Deeper	4.29	0.952
Adult	Control	NeuN	Upper	1.61	0.121
			Middle	1.56	0.185
			Deeper	1.60	0.167
	FCDIIB	NeuN only	Upper	1.52	0.194
			Middle	2.61	0.511
			Deeper	1.06	0.277
	FCDIIB	SMI32 (+)	Upper	2.22	0.846
			Middle	5.18	1.21
			Deeper	3	0.759

FCD = focal cortical dysplasia; SEM = standard error of the mean.

## Discussion

FCD type 2b is a common cause of pharmaco-resistant epilepsy, characterized by abnormal cortical layering, cytological abnormalities and aberrant functional properties of dysmorphic neurons.^1,3^ In addition, the activity of morphologically unaffected, surrounding neurons is profoundly altered, resulting in hyperexcitable, maldeveloped networks within lesions and the perilesional microenvironment.^6,39-42^ Since DA input to cortical areas is known to modulate signal-to-noise ratio and excitation/inhibition (E/I) balance in local cortical networks,^14,43,44^ alterations in this monoaminergic input may also contribute to the hyperexcitability in the lesion areas. To investigate this possibility, we examined DA innervation in human FCD type 2b biopsies and in a mouse model of FCD type 2 and provide evidence for altered DA innervation and varicosity density, aberrant DA innervation patterns and abnormal dopamine receptor expression in neuronal dysplasia elements in FCD type 2b areas. Our results provide insight into alterations in DA innervation and receptor expression in FCD type 2b and suggest that resulting changes in DA neurotransmission in FCD type 2b areas may be involved in abnormal network activity.

Our analysis shows that at adult stages, DA innervation density is decreased in p.Leu2427Pro mice and FCD type 2b areas compared with control animals or control areas, respectively, while varicosity density is comparable in FCD type 2b and control areas. In contrast, we did not find any significant changes in DA innervation density between WT-mTOR and p.Leu2427Pro mice, or between control and FCD type 2b areas at adolescent stages, but a trend towards decreased density of varicosities in FCD type 2b areas (Figs 1 and 4). During adolescence, DA axons in the cortex undergo extensive remodelling and DA innervation density increases until early adulthood in rodents and primates.^45^ In our mouse model, we indeed find that in control mice innervation density increases significantly between the adolescent and adult stage. In contrast, there is no further increase in innervation density in the mPFC of p.Leu2427Pro mice. These results suggest that alterations in early cortical development and the resulting cortical malformations begin to affect DA innervation during adolescence but alterations only fully manifest in adulthood. The mechanisms that regulate the maturation of DA innervation in cortical areas are not well understood. Thus, it remains to be investigated how cortical malformations impact on the maturation of DA axon density. Molecular changes (e.g. changes in guidance cues such as netrin), altered synaptic pruning due to changes in local microglia activity or a direct impact of functional alterations in the local networks on DA fibres in the FCD type 2b areas could play a role.^45,46^

The formation of varicosities on DA fibres, which act as potential DA release sites, is likely an important indicator of functional DA fibre maturation. Varicosities on DA axons are found adjacent to both pyramidal and non-pyramidal somata in rodent and primate PFC.^47,48^ The number of DA appositions onto neuronal cell bodies increases steadily from neonatal to adult stages.^45^ The increase in DA fibre and varicosity density coincides with a steady increase in dopamine tissue content between early (P25) and late adolescence (P45) in rats and a sharp increase between late adolescence and adulthood.^37^ Similarly, studies in non-human primate PFC indicate an increase in DA innervation during adolescence, with a peak observed in 2–3-year old adolescent animals,^49,50^ coinciding with a higher dopamine tissue concentration compared with younger ages.^51^ Taken together, these results suggest that a reduced density of DA varicosities in FCD type 2b areas during adolescence and a reduced DA innervation density in FCD type 2b areas in the adult cortex may result in reduced dopamine levels in the affected areas.

In the context of neurotransmitter release, it should also be taken into consideration that a subset of DA inputs to the PFC are capable of co-releasing glutamate.^52^ Thus, a reduction in DA innervation and varicosity density in FCD type 2b areas could affect both local dopamine and glutamate release and the combined reduction of both neurotransmitters could ultimately alter local network activity. Indeed, we and others have already shown that stimulation of DA inputs in the PFC leads to glutamate-mediated excitation of fast-spiking interneurons and, to a lesser extent, of pyramidal neurons.^53-55^

The impact of dopamine on local networks does not only depend on its release but also on the cell-type-specific expression of dopamine receptors. Using a FISH approach that allows quantitative analysis of *Drd1*/*DRD1* and *Drd2*/*DRD2* transcript expression in both mouse mPFC and human specimens, we show that dopamine receptor transcripts are specifically elevated in mTOR mutant (p.Leu2427Pro) neurons in the mouse model (in both adolescents and adults) and in dysmorphic neurons in human FCD 2b areas (in paediatric patients and the frontal cortex of adult patients), but not in surrounding neurons (Figs 2 and 5). This specific upregulation of *Drd1*/*DRD1* and *Drd2*/*DRD2* in dysmorphic/mTOR mutant neurons during adolescence raises intriguing questions on the underlying mechanisms. One possibility is that the increased expression of dopamine receptors is simply a consequence of the on average larger cell size of mTOR mutant/dysmorphic neurons.^56^ However, we did not find a significant correlation between *Drd1*/*DRD1* and *Drd2*/*DRD2* expression and size of the cell soma (Supplementary Figs 5E, F, 6E, F and 9). Another explanation could be that analogous to the consequences of *Tsc1* inactivation in neural precursors during embryonal development,^57^ mTOR mutant/dysmorphic neurons possess higher plasticity than surrounding neurons, enabling them to compensate for potentially diminished dopamine levels by upregulating dopamine receptors. Alternatively, this phenomenon may be intrinsic to the mutant neurons, with *Drd* upregulation occurring independently of the reduced DA innervation. On a functional level, the increased expression of dopamine receptors in the mTOR-mutant/dysmorphic neurons may disrupt the proper integration of excitatory and inhibitory inputs in these neurons,^18^ potentially contributing to aberrant neuronal activity.

As discussed earlier, altered DA neurotransmission is a likely result of the changes in DA innervation, varicosity density and dopamine receptor expression that we observe in FCD type 2b areas/FCD type 2 mouse model. The altered DA neurotransmission could aggravate abnormal activity in the lesion by having effects on both the E/I balance and the signal-to-noise ratio of local network activity in the adult brain^14,58^ and the maturation of local network activity. DA activity has been shown to influence striatal medium spiny neurons and PFC pyramidal neuron maturation during a critical period.^59-61^ Complete absence of DA innervation in the murine PFC throughout development results in deficient maturation of parvalbumin- and calbindin-expressing PFC interneurons. These deficits only become apparent during adolescence, and some persist into adulthood.^62^ On the other hand, phasic stimulation of DA neuron activity induces mesofrontal circuit plasticity during adolescence and importantly, frontal DA neurostimulation during adolescence has the potential to reverse cognitive deficits associated with genetic models of schizophrenia.^38,63,64^ These data highlight the crucial role of DA signalling in shaping cortical circuits during the critical period of adolescence and point towards the possibility of using targeted and developmental-stage-specific modulation of dopamine release to ameliorate aberrant activity of local cortical networks.

### Limitations of the study

The number of patients each from paediatric and adult FCDs is limited with certain heterogeneity and factors including gender differences in dopamine receptors may be relevant.^65^ However, intra-individual comparisons of data from control versus lesion areas as we applied them here should minimize these effects. Sampling of diseased versus control samples is another critical topic in studies dealing with human epilepsy surgical brain tissue. Combinations of MRI, invasive EEG analysis and histological sampling to differentiate ‘control’ from ‘epileptic’ tissue samples have been recently applied.^66,67^ These complex procedures may be particularly helpful in the context of epileptogenic lesions, where the epileptogenic zone is difficult to determine by neuropathological means. A combination of stereological EEG and histology observed that the epileptogenic zone corresponded to histologically defined FCD in the vast majority of cases.^68^ Furthermore, traumatic tissue damage as induced by EEG electrode implantation may lead to DA plasticity. Traumatic insults have been demonstrated to alter dopamine receptor expression in brain tissue.^69^ These data may support our sampling strategy in the present study, which focuses on DA innervation in the neuropathological lesion versus non-lesion regularly structured brain tissue as ‘control’ despite the fact that tissue sampling from epilepsy surgery for experimental research is a matter of intense ongoing debate. Finally, our present study applies state-of-the-art molecular neuroanatomical and pathological approaches in strictly stratified human neurosurgical biopsies and in a complementary mouse model of FCD. Electrophysiological experiments addressing changes in the DA input to FCDs in association with enhanced cortical excitability in the form of epileptic discharges and seizures represents an intriguing task for the future.

## Conclusion

Our results on altered DA axonal innervation, varicosity density and dopamine receptor expression in FCD type 2b areas suggest impaired DA neurotransmission in FCD type 2b lesions and provide novel insights into the pathophysiology of cortical malformations in FCD type 2b patients. Our findings emphasize the necessity for further investigation into the role of long-range modulatory inputs and dopamine neurotransmission in the pathogenesis of FCD type 2b and their potential function in modulating epileptic manifestations in affected patients.

## Supplementary Material

awaf080_Supplementary_Data

## Data Availability

The data that support the findings of this study are available from the corresponding author, upon reasonable request.
