# Supplementary material for: Alterations in dopaminergic innervation and receptors in focal cortical dysplasia
Source: Brain. 2025 Apr 16;148(8):2899–911. doi: 10.1093/brain/awaf080 (PMC12316006; doi:10.1093/brain/awaf080)
Supplement: awaf080_Supplementary_Data [file awaf080_supplementary_data.zip › Author contribution.pdf]

Author contributions

N.M.: data curation, formal analysis, validation, investigation, visualization, methodology, software, writing—original draft, writing—review and editing; K.S.: software, formal analysis, writing—review and editing; J.P.: methodology, writing—review and editing; S.K.: supervision, writing—review and editing; V.B.: resources, T.B.: resources; A.B.: conceptualization, resources, data curation, supervision, funding acquisition, validation, methodology, project administration, writing—review and editing.; S. B.: conceptualization, resources, data curation, formal analysis, supervision, funding acquisition, validation, methodology, project administration, writing—review and editing.
