# Supplementary material for: Alterations in dopaminergic innervation and receptors in focal cortical dysplasia
Source: Brain. 2025 Apr 16;148(8):2899–911. doi: 10.1093/brain/awaf080 (PMC12316006; doi:10.1093/brain/awaf080)
Supplement: awaf080_Supplementary_Data [file awaf080_supplementary_data.zip › Supplementary data.pdf]

Supplementary Information

Alterations in dopaminergic innervation and receptor expression in mouse models and patients with Focal Cortical Dysplasia

Norisa Meli[1,2], Katherine Sheran[3], Julika Pitsch[4], Sabine Krabbe[3], Valeri Borger[5], Tobias Baumgartner[4], Albert Becker[2], Sandra Blaess[1]

1 Neurodevelopmental Genetics, Institute of Reconstructive Neurobiology, Medical Faculty, University of Bonn, Bonn, Germany

2 Institute for Cellular Neurosciences II, Medical Faculty, University of Bonn, Bonn, Germany

3 German Center for Neurodegenerative Diseases (DZNE), Bonn, Germany

4 Department of Epileptology, University Hospital Bonn, Bonn, Germany

5 Department of Neurosurgery, University Hospital Bonn, Bonn, Germany

*Correspondence to: sandra.blaess@uni-bonn.de & albert_becker@uni-bonn.de

Supplementary Figure 1 Overview of electroporated neurons and overall comparison of neuron volume between different cell types in the mouse mPFC. A, B) IUE validation in (**A)** WT-mTOR and **(B)** p.Leu2427Pro mPFC at P30. **C, D)** IUE validation in (**C)** WT-mTOR and **(D)** p.Leu2427Pro mPFC at P60. Scale bar: 1000 µm. **E, F)** Comparison of mean volume for each neuronal group in the **(E)** upper and **(F)** deeper layers of the mPFC at P30. The data points in the boxplots represent the mean neuronal volume for each biological replicate, while the median value is indicated by the horizontal line within the boxplot. Upper layers: Two-way ANOVA with repeated measures followed by Bonferroni test to correct for multiple comparisons between the indicated groups. Deeper layers: paired t-test to compare GFP (-) and GFP (+) groups within the p.Leu2427Pro subjects and unpaired t-test for the other indicated comparisons. P-values were adjusted for multiple comparisons using the Bonferroni-Dunn method. n = 3 WT-mTOR and 2 p.Leu2427Pro mice. **G, H)** Distribution of neuronal volume for each neuronal group of the **(G)** upper or **(H)** deeper layers of the WT-mTOR or p.Leu2427Pro mPFC at P30. **I, J)** Comparison of mean volume for each neuronal group in the **(I)** upper and **(J)** deeper layers of the mPFC at P60. Upper layers: Two-way ANOVA with repeated measures followed by Bonferroni test to correct for multiple comparisons between the indicated groups. Deeper layers: paired t-test to compare GFP (-) vs. GFP (+) groups within the p.Leu2427Pro experimental group and unpaired t-test for the other indicated comparisons. P-values were adjusted for multiple comparisons using the Bonferroni-Dunn method. n = 4 mice per group. **K, L)** Distribution of neuronal volume for each neuronal group of the **(K)** upper or **(L)** deeper layers of the WT-mTOR or p.Leu2427Pro mPFC at P60. Groups in WT-mTOR mPFC: RFP (-): non-electroporated, NeuN+ neurons; RFP (+): neurons electroporated with WT-mTOR plasmid, NeuN+. Groups in p.Leu2427Pro mPFC: GFP (-): non-electroporated, NeuN+ neurons; GFP (+): neurons electroporated with mTOR-p.Leu2427Pro plasmid, NeuN+ . See Figure 2 for a detailed description of groups of neurons analyzed.

Supplementary Figure 2 Phospho-S6 (Ser240/244) expression in mouse mPFC at P30. A, B) Representative images of pS6 expression in the **(A)** WT-mTOR and **(B)** p.Leu2427Pro mPFC. Scale bar: 200 µm. Smaller panels: higher magnifications of the indicated boxed areas from the whole mPFC images. Scale bar: 20 µm. **(C)** Quantification of the pS6 mean intensity expression within RFP+/GFP+ neurons and non-electroporated (RFP-/GFP-) neurons in WT-mTOR and p.Leu2427Pro mPFCs. n = 3 mice per group. Two-way ANOVA with repeated measures followed by Bonferroni test to correct for multiple comparisons between the

indicated groups. The data points in the boxplots represent the mean grey values of each biological replicate, while the median value is indicated by the horizontal line within the boxplot.

Supplementary Figure 3 Quantification of TH and NET colocalization in the mouse mPFC. (A) Representative images of axons labelled with TH and NET in the upper layer of the mPFC. Arrows indicate fibers in which the markers are colocalized. Scale bar: 20 μm. (**B)** Quantification of colocalization of TH and NET in axons in the upper and deeper layers of the mPFC. Percent colocalization indicates the percentage of the overlap (TH+ & NET+ area)/total TH+ area. *n* = 3, P45. **(C)** NET axonal analysis in the mouse mPFC at P30. *n* = 5 WT-mTOR vs. 3 p.Leu2427Pro mice, t-test. Occupation index is calculated as the percentage of the area covered by axons in each sub-region divided by the total area of that sub-region. The data points in the boxplots represent the mean occupational index for each biological replicate, while the median value is indicated by the horizontal line within the boxplot.

Supplementary Figure 4 Approach to divide the mPFC into layers and sub-regions and altered density of dopaminergic (DA) innervation in subregions of the mPFC of the p.Leu2427Pro mTOR mouse model. (A) Overview of an mPFC mouse section matched with the corresponding atlas level from the Allen Brain Reference Atlas (http://atlas.brain-map.org). **(B)** Defining the upper (I & II/III) and deeper (V & VI) layers and areas of the mouse mPFC at P30 based on DAPI staining and the corresponding level of the Allen Brain Reference Atlas. (**C)** RFP+ IUE-targeted neurons are located in the upper layers defined by the approach shown in A and B (mainly layer II/III), validating this method of layer definition. (**D)** Representative image of p.Leu2427Pro mouse model immunostained for TH, GFP and RFP (also shown in Figure 1A) depicting the divisions into mPFC subregions: anterior cingulate (AC), prelimbic (PL), and infralimbic cortex (IL). **E, F)** Quantification of DA axonal density in the subregions of the mouse mPFC at P30 (*n* = 7) **(E)** and P60 (*n* = 5) **(F)**. T-test was performed between WT-mTOR and p.Leu2427Pro for each respective sub-region, except for P30 upper layer where Mann-Whitney-Wilcoxon test was performed instead. Occupation index is calculated as the percentage of the area covered by axons in each sub-region divided by the total area of that sub-region. The data points in the boxplots represent the mean occupational index for each biological replicate, while the median value is indicated by the horizontal line within the boxplot. Scale bars: 200 μm.

Supplementary Figure 5 Additional comparison of *Drd1* and *Drd2* mRNA receptor expression in the P30 mouse mPFC.

A, C) Distribution of puncta/cell quantifications of **(A)** *Drd1* and **(C)** *Drd2* mRNA expression after pooling all analyzed neurons of the upper or deeper layers of the WT-mTOR or p.Leu2427Pro mPFC at P30. Violin plots depict the distribution of quantified mRNA expressions and inner boxplots show the median expression (horizontal line) for each neuron group. See Figure 2 for a detailed description of groups and number of neurons analyzed. **B, D)** Mean expression of **(B)** *Drd1* and **(D)** *Drd2* mRNA transcripts shown only for the GFP (+) groups from both upper and deeper layers of WT-mTOR and p.Leu2427Pro mPFC. The data points in the boxplots represent the mean puncta/cell expression for each biological replicate, while the median value is indicated by the horizontal line within the boxplot. $n = 3$ mice per group, unpaired t-test. See Figure 2 for a detailed description of groups and number of neurons analyzed. **E, F)** Correlation analysis between cell volume and **(E)** *Drd1* or **(F)** *Drd2* mRNA receptor expression in the mouse mPFC. Spearman correlation coefficients and p values calculated for each cell type. $n = 3$ WT-mTOR vs. 2 p.Leu2427Pro mice.

Supplementary Figure 6 Additional comparison of *Drd1* and *Drd2* mRNA receptor expression in the P60 mouse mPFC.

A, C) Distribution of puncta/cell quantifications of **(A)** *Drd1* and **(C)** *Drd2* mRNA expression after pooling all analyzed neurons of the upper or deeper layers of the WT-mTOR or p.Leu2427Pro mPFC at P60. Violin plots depict the distribution of quantified mRNA expressions and inner boxplots show the median expression (horizontal line) for each neuron group. See Figure 2 for a detailed description of groups and number of neurons analyzed. **B, D)** Mean expression of **(B)** *Drd1* and **(D)** *Drd2* mRNA transcripts shown only for the GFP (+) groups from both upper and deeper layers of WT-mTOR and p.Leu2427Pro mPFC. The data points in the boxplots represent the mean puncta/cell expression for each biological replicate, while the median value is indicated by the horizontal line within the boxplot. $n = 4$ mice per group, unpaired t-test. See Figure 2 for a detailed description of groups and number of neurons analyzed. **E, F)** Correlation analysis between cell volume and **(E)** *Drd1* or **(F)** *Drd2* mRNA receptor expression in the mouse mPFC. Spearman correlation coefficients and p values calculated for each cell type. $n = 4$ mice per group.

Supplementary Figure 7 Layer distinction in control and FCD type 2b areas in human specimen. A, B) Immunhistochemistry performed for NeuN, Calretinin and SMI32 to define cortical layers in **(A)** control and **(B)** FCD type 2b areas. NeuN labels all neurons and helps to visualize the cortical layers. Calretinin is expressed in supragranular interneurons predominantly in layer II. Expression is more dispersed in layer III. Calretinin in combination with NeuN helps to distinguish upper (Layer I and II) from middle (Layer III & IV) layer. SMI32 is highly expressed in neuronal processes in layers III and V.[32] This aids in discriminating middle from deeper (Layer V & VI) layers. Example from a paediatric patient. Scale bars: 500 µm.

Supplementary Figure 8 *DRD1* and *DRD2* expression in upper, middle and deeper layers of control and FCD type 2b areas in paediatric and adult patients.
A, B) Distribution of puncta/cell quantifications of *DRD1* and *DRD2* mRNA transcripts after pooling all analyzed cells in **(A)** paediatric and **(B)** adult human specimen. Violin plots depict the distribution of quantified mRNA expressions and inner boxplots show the median expression (horizontal line) for each neuron group. **(C)** Distribution of expression levels (puncta/cell) in paediatric human FCD type 2b (FCDIIB) specimen per layer. Quantified cells for *DRD1*: upper layers: 31 NeuN, 8 NeuN only, 2 NeuN SMI32 (+), middle layers: 40 NeuN, 12 NeuN only, 13 NeuN SMI32(+), deeper layers: 34 NeuN, 19 NeuN only, 11 NeuN SMI32 (+). Quantified cells for *DRD2*: upper layers: 40 NeuN, 8 NeuN only, 4 NeuN SMI32 (+), middle layers: 34 NeuN, 13 NeuN only, 13 NeuN SMI32(+), deeper layers: 36 NeuN, 16 NeuN only, 14 NeuN SMI32 (+). **(D)** Distribution of expression levels (puncta/cell) in adult human FCD type 2b specimen. Quantified cells for *DRD1*: upper layers: 82 NeuN, 31 NeuN only, 13 NeuN SMI32 (+), middle layers: 59 NeuN, 36 NeuN only, 20 NeuN SMI32(+), deeper layers: 72 NeuN, 34 NeuN only, 9 NeuN SMI32 (+).Quantified cells for *DRD2*: upper layers: 126 NeuN, 50 NeuN only, 9 NeuN SMI32 (+), middle layers: 75 NeuN, 33 NeuN only, 17 NeuN SMI32(+), deeper layers: 88 NeuN, 17 NeuN only, 12 NeuN SMI32. n = 3 specimen for paediatric and for adult patients. The data signs in the boxplots represent the puncta/cell expression for each individual neuron, while the median value of the group is indicated by the horizontal line within the boxplot. Symbols: ▼▲frontal cortex, ●parietal cortex.

Supplementary Figure 9 Correlation analysis of cell soma size and *DRD1* and *DRD2* puncta quantification in (A) paediatric and (B) adult human specimen. Spearman

correlation coefficients and p values calculated for each cell type. *n* = 3 specimen for paediatric and for adult patients. See Figure 5 for a detailed description of groups and number of neurons analyzed.

Supplementary Figure 1

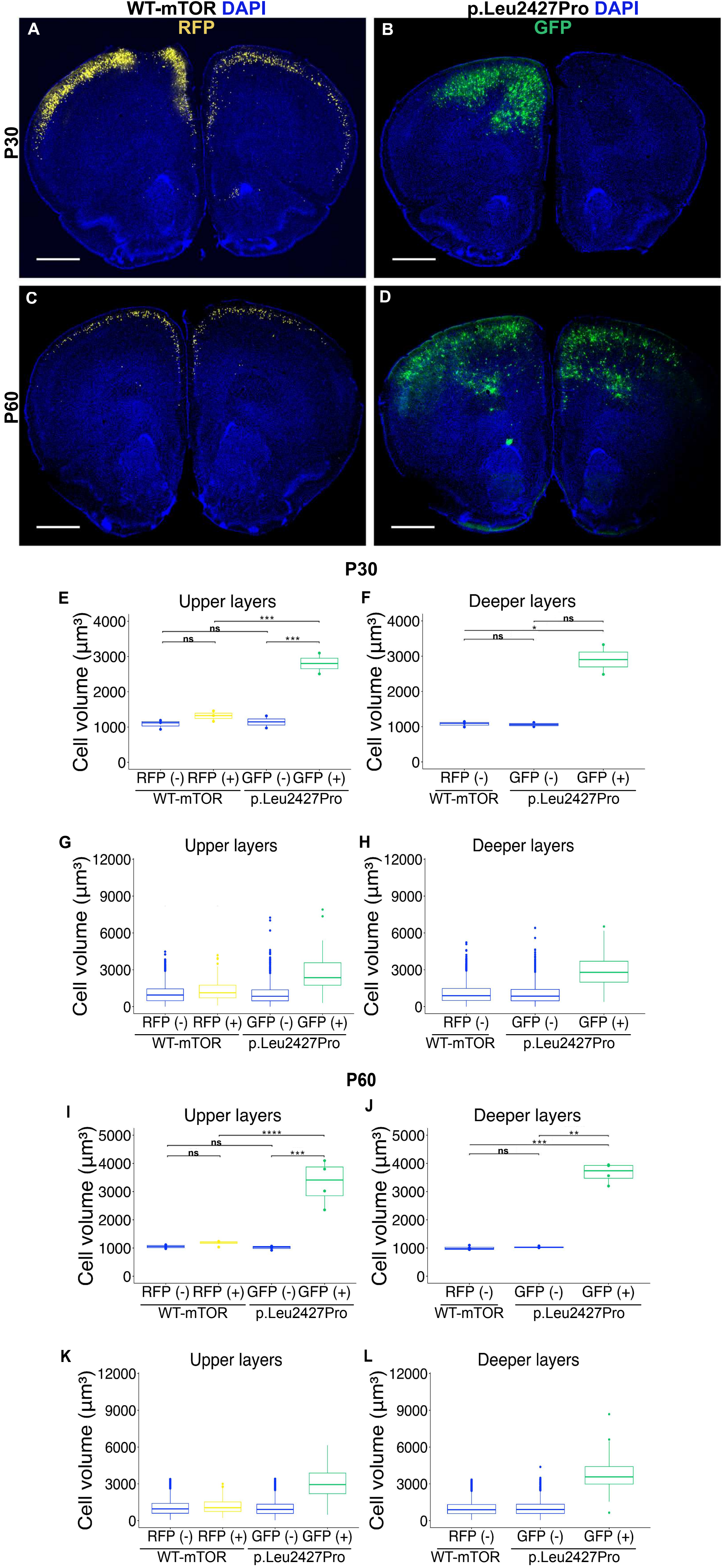

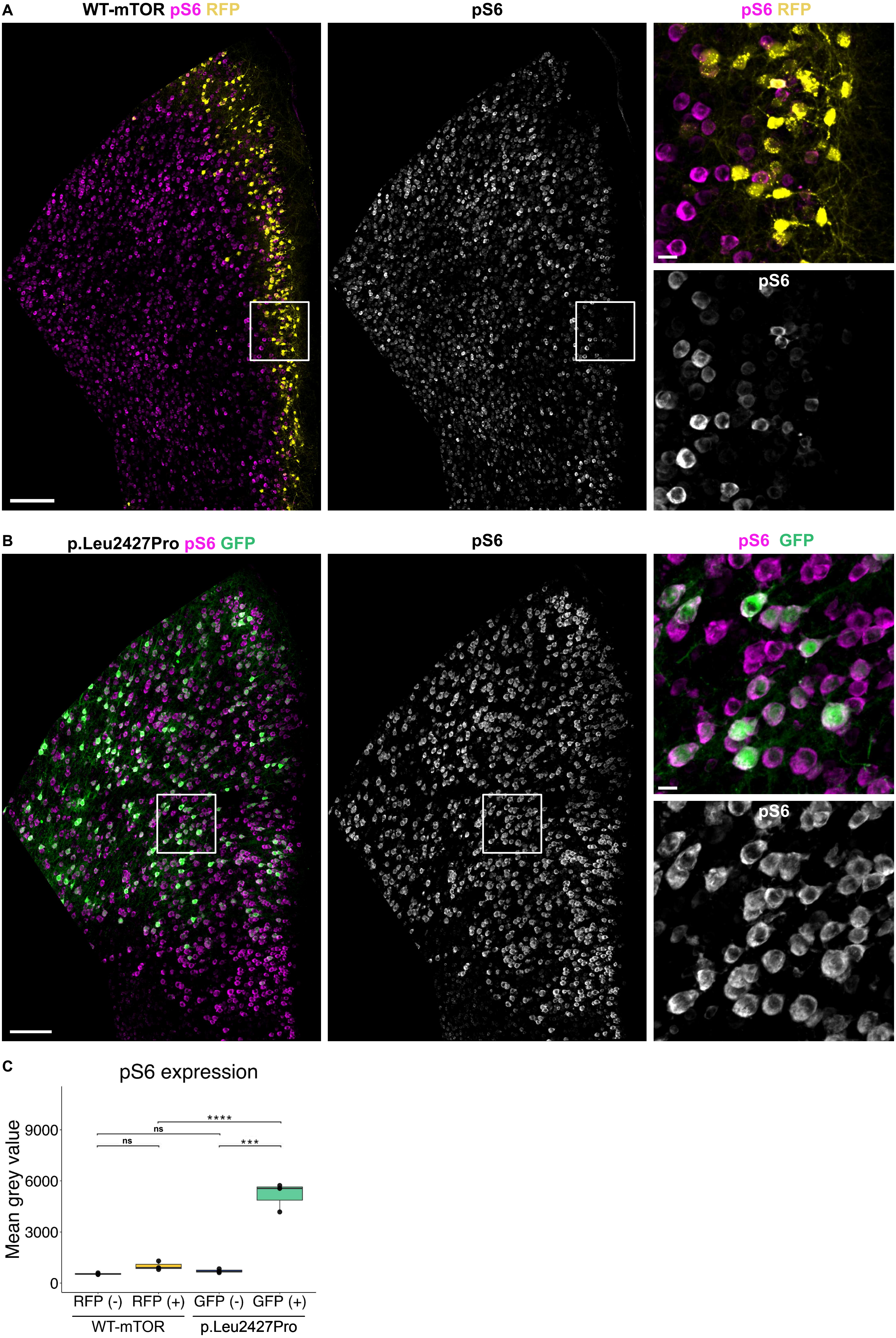

Supplementary Figure 2

Supplementary Figure 3

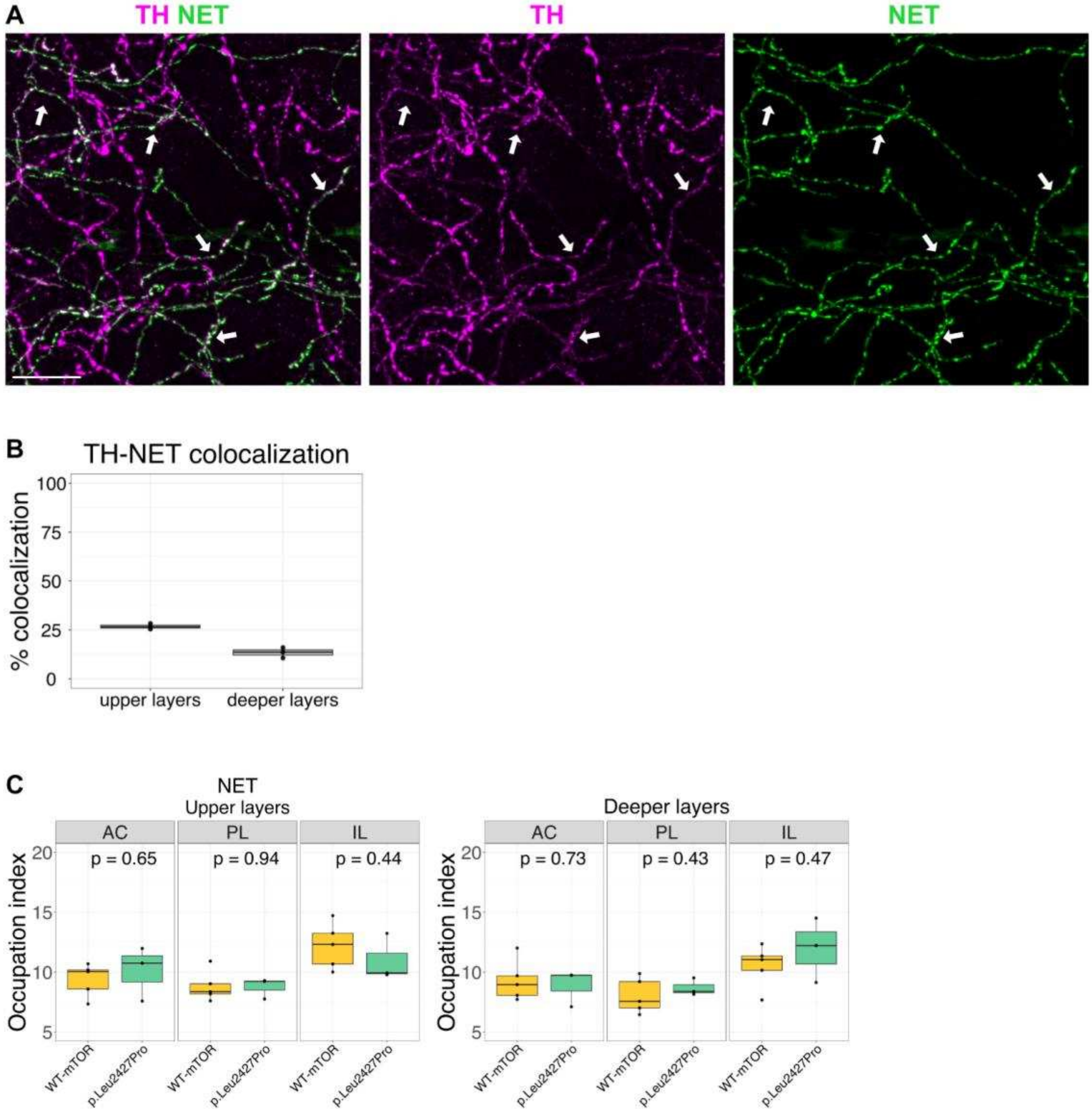

Supplementary Figure 4

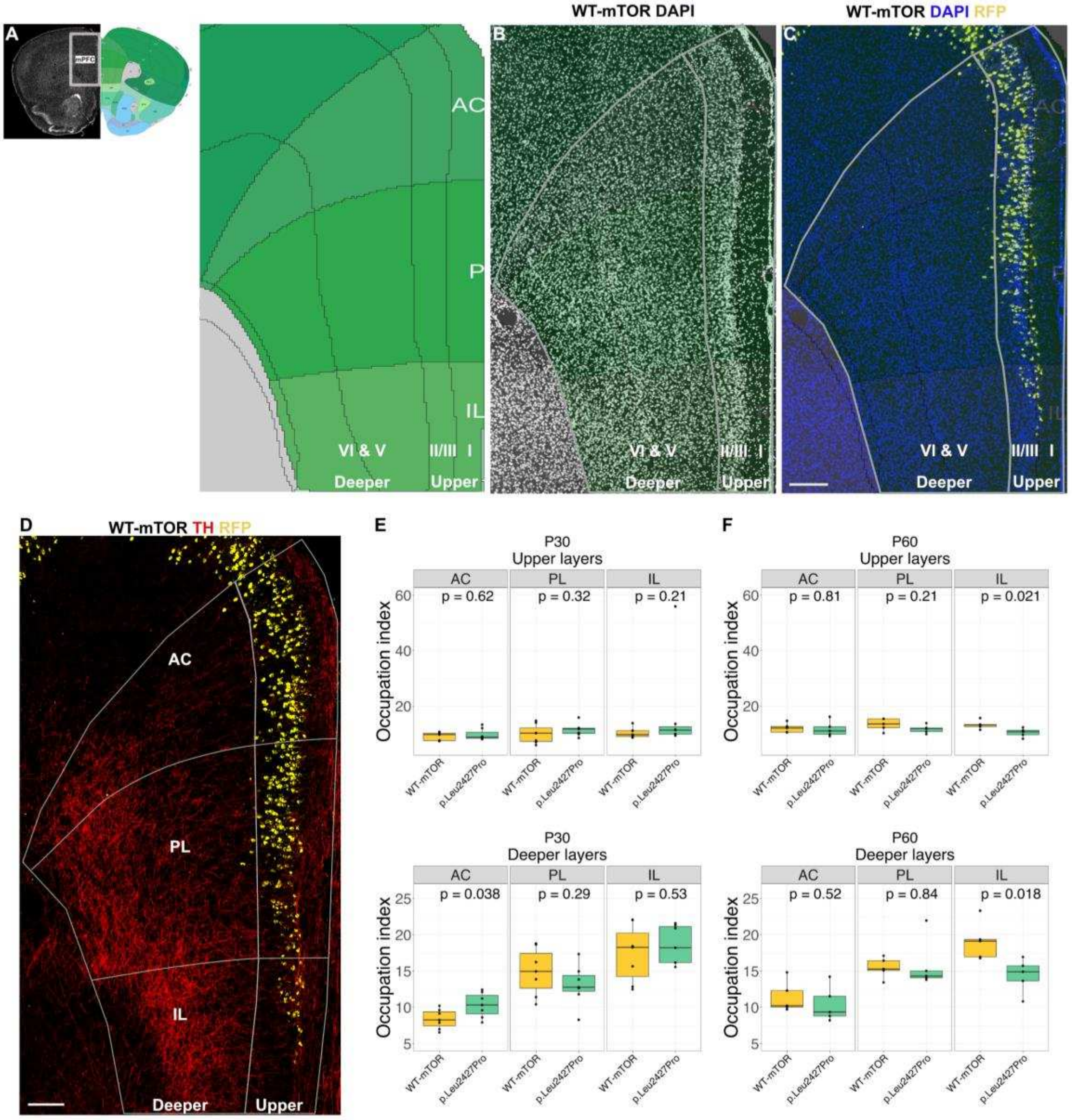

Supplementary Figure 5

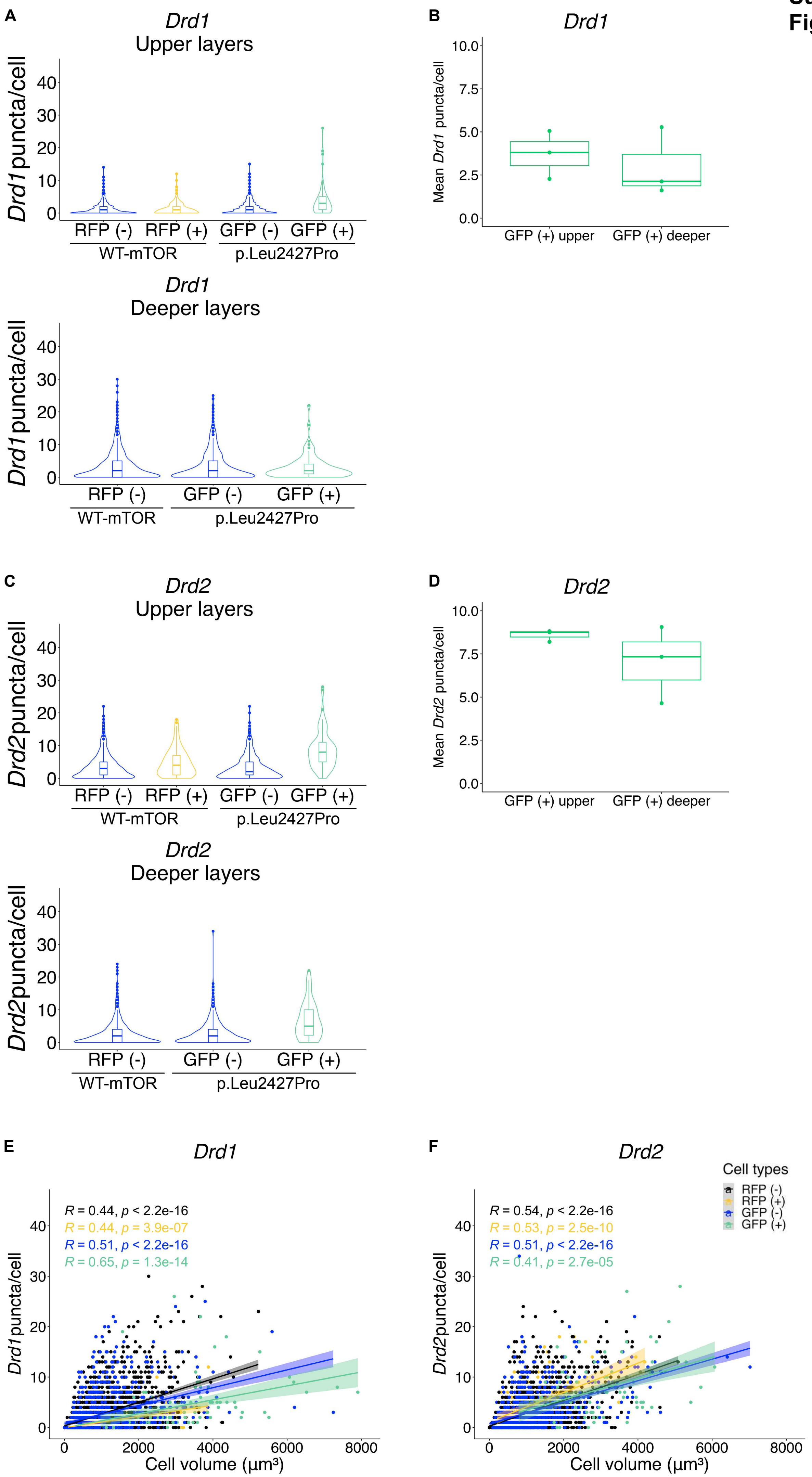

Supplementary Figure 6

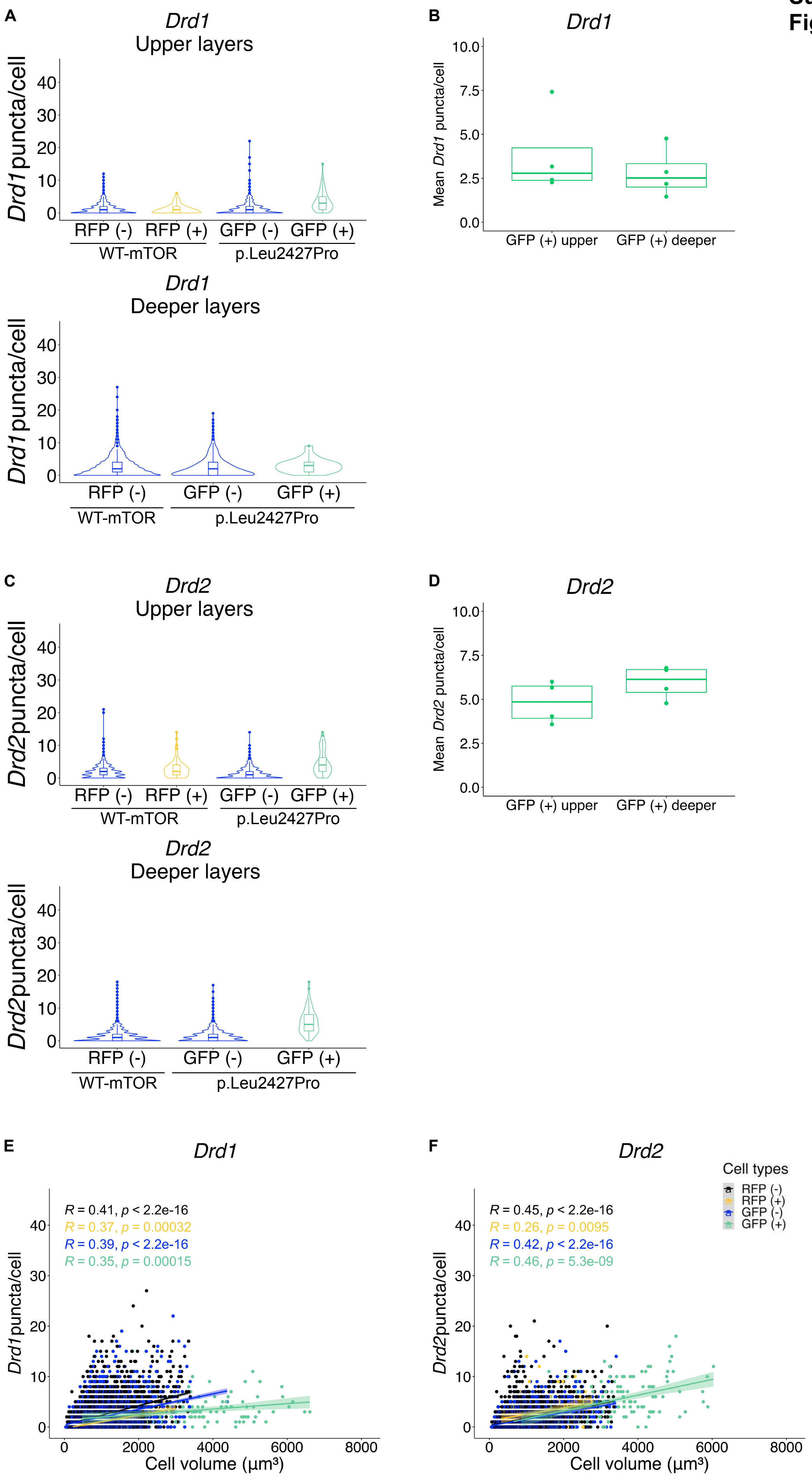

Supplementary Figure 7

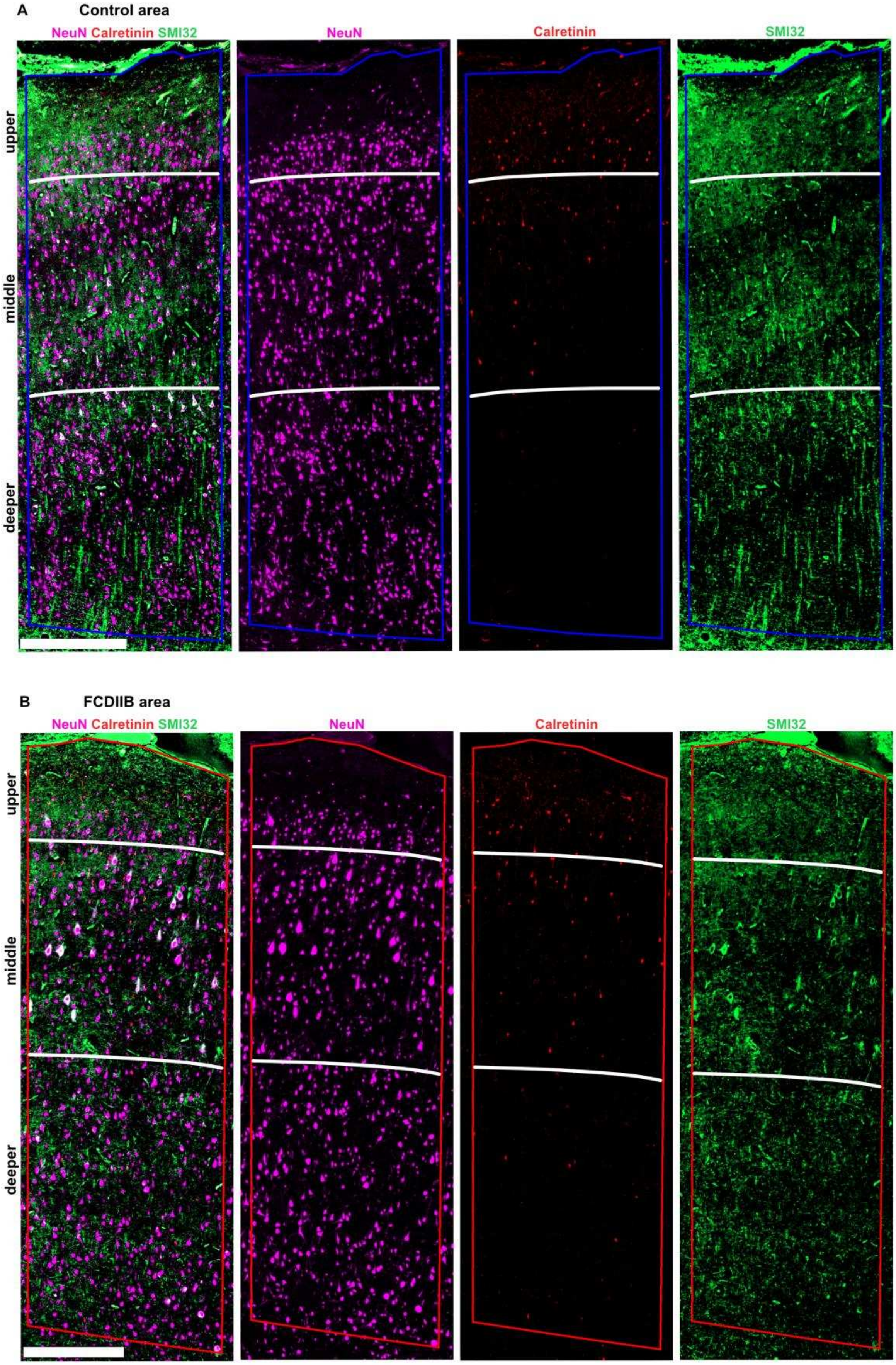

Supplementary Figure 8

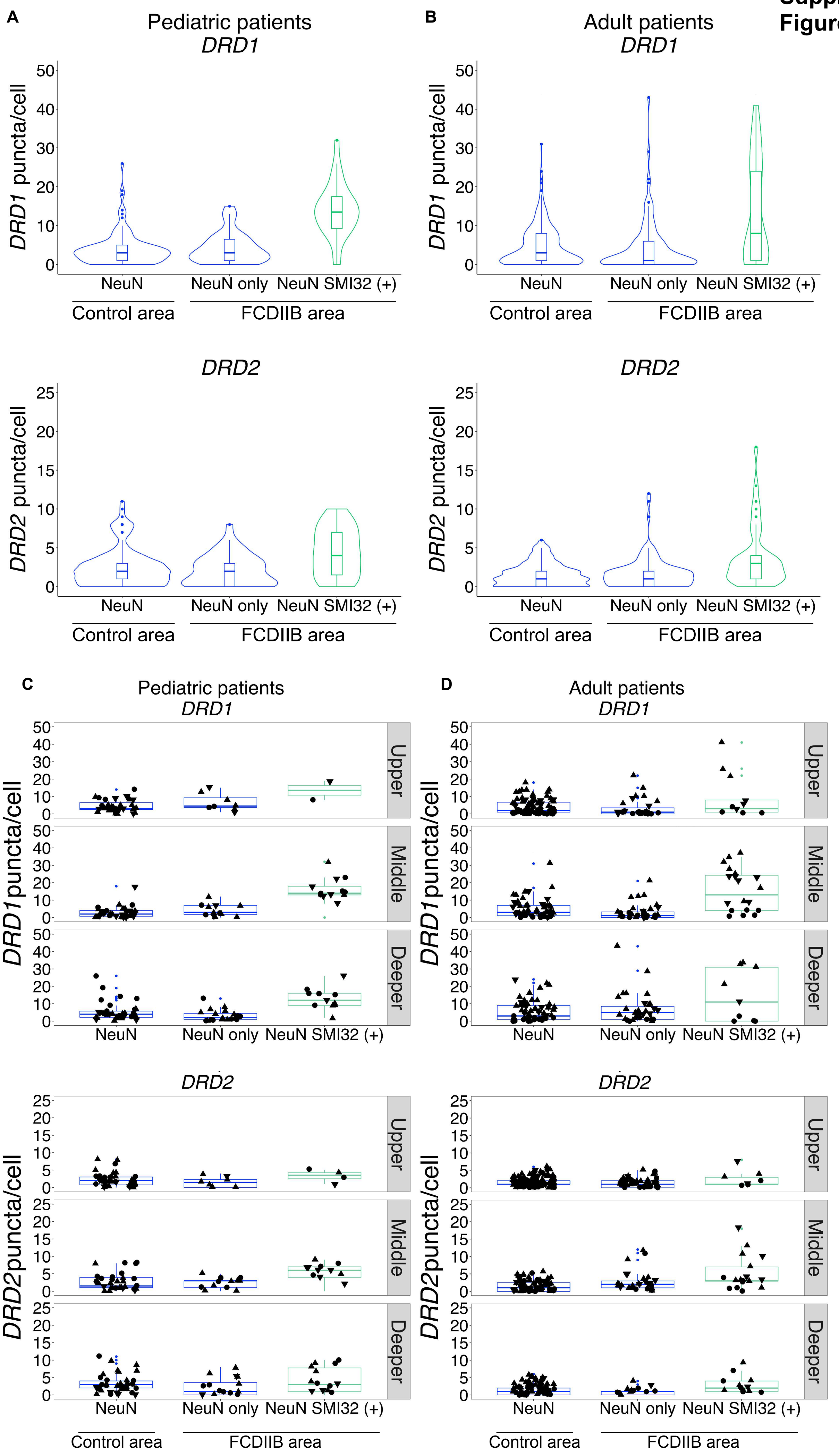

Supplementary Figure 9

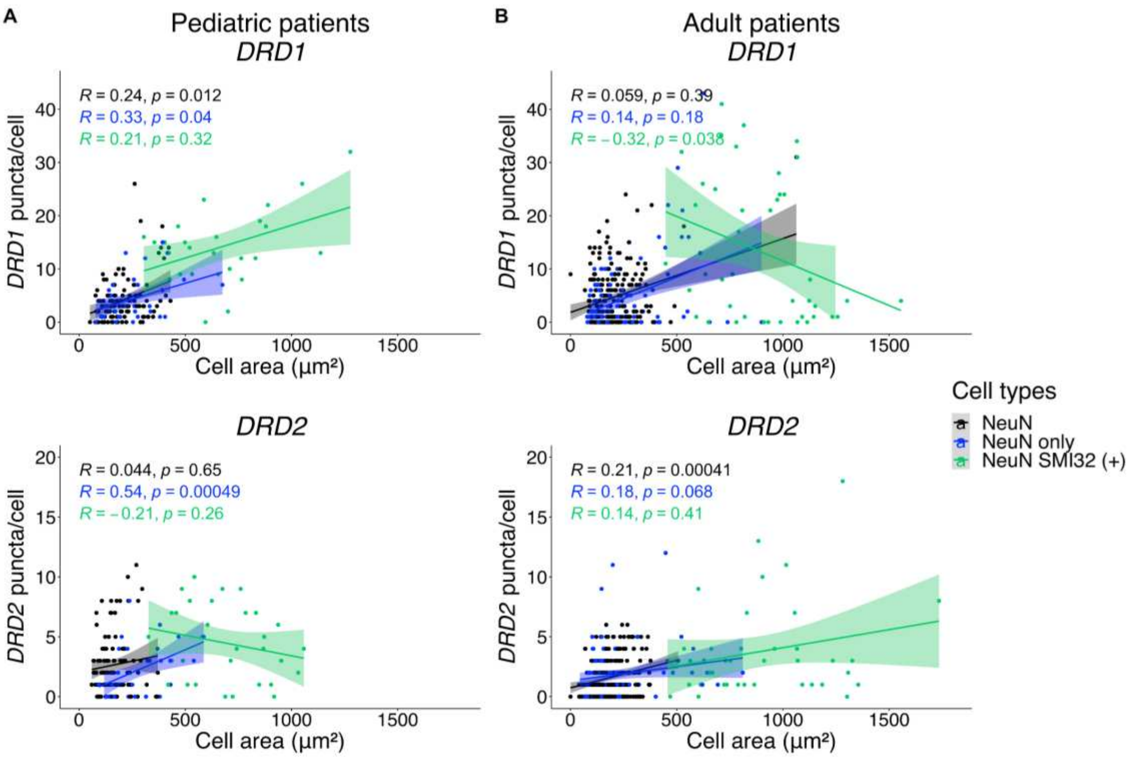

Supplementary table 1 List of primary and secondary antibodies

Antibody	Host species - Target	Dilution	Catalog no.	Manufacturer
TH	rabbit	1:500	AB152	Merck Millipore
NeuN	rabbit	1:500	ab177487	Abcam
NeuN	guinea pig	1:500	266004	Synaptic Systems
Neurofilament H (SMI32)	mouse	1:1000	801702	BioLegend
Calretinin	goat	1:1000	CG1	Swant
GFP	rat	1:1000	04404-84	Nacalai Tesque, Inc
RFP	rat	1:1000	5f8-20	ChromoTek
Phospho-S6 Ribosomal Protein (Ser240/244)	rabbit	1:1000	5364	Cell Signaling Technology
NET (SLC6A2)	mouse	1:500	AMAB91116	Atlas antibodies
Alexa 647	donkey - rabbit	1:500	A31573	Life Technologies
Alexa 647	donkey - guinea pig	1:500	706-605-148	Jackson ImmunoResearch
Alexa 546	donkey - rabbit	1:500	A10040	Life Technologies
Alexa 488	donkey - mouse	1:500	A21202	Life Technologies
Alexa 488	donkey - rat	1:500	A21208	Life Technologies
Alexa 405	donkey - rabbit	1:500	ab175649	Abcam
biotin	donkey - goat	1:200	705-065-147	Jackson ImmunoResearch
biotin	donkey - rabbit	1:200	711-065-152	Jackson ImmunoResearch
Cy3	donkey - rat	1:200	712-165-153	Jackson ImmunoResearch
Cy3-Streptavidin	- biotin	1:1000	016-160-084	Jackson ImmunoResearch
TSA Vivid 650	- RNAscope® probes	1:3000	7527	Tocris
