# Supplementary material for: Alterations in dopaminergic innervation and receptors in focal cortical dysplasia
Source: Brain. 2025 Apr 16;148(8):2899–911. doi: 10.1093/brain/awaf080 (PMC12316006; doi:10.1093/brain/awaf080)
Supplement: awaf080_Supplementary_Data [file awaf080_supplementary_data.zip › Supplementary Material.pdf]

Processing of mouse tissue

Postnatal day (P) 30 and P60 mice were terminally anesthetized and transcardially perfused with PBS followed by 4% paraformaldehyde (PFA). Brains were extracted and post-fixed with 4% PFA overnight and stored at -80°C until cryosectioned. Brains were sectioned coronally at 40 µm thickness and stored as free-floating sections in an anti-freeze solution at -20°C.

Processing of human tissue

Human FCD type 2b patient biopsy samples diagnosed based on the current International League Against Epilepsy (ILAE) classification[1] were obtained during surgical resections at the University of Bonn Medical Center Neurosurgery Program with patient consent and ethical approval (nr. 308/19) and were processed for formalin-fixation paraffin-embedding (FFPE). FFPE biopsies were sectioned into 4 µm serial sections and Hematoxylin and Eosin staining was used to define control and FCD area. Subsequently, immunostaining was performed to define cortical layers (NeuN, Calretinin and SMI32) and dysmorphic neurons (SMI32). NeuN serves as a general neuronal marker, Calretinin is expressed in layer II/III neurons and SMI32 (also known as Neurofilament H) - in addition to its expression in the soma of dysmorphic neurons - is highly expressed in neuronal processes in layer III and V.[2] Then, a set of up to 10 serial sections was immunostained with tyrosine hydroxylase (TH) to mark DA axons. In addition, a set of sections was used to analyze *DRD1/2* transcript expression using FISH.

Imaging

Mouse mPFC sections utilized for DA and NA axonal analysis, *Drd1/Drd2* FISH analysis, TH/NET colocalization analysis and human FCD 2b sections processed for *DRD1/DRD2* FISH were imaged with the Visitron VisiScope Confocal Spinning Disk Microscope (UKB Microscope Core Facility). First, a 5x air objective was utilized to take an overview image of the whole section. Afterwards, a 40x water immersion objective was used to image the region of interests with laser lines 405 nm, 488 nm/561 nm and 640 nm for mouse mPFC sections and 488 nm, 561 nm and 640 nm for human FCD 2b sections. To achieve full area coverage and maximum focus, tile regions

and z-stacks with 2 µm step/each were acquired, respectively. Tiled images were stitched with the Visitron Software.

Human FCD 2b serial sections processed for TH, NeuN and cortical marker IHC were imaged with Zeiss AxioScan.Z1 Widefield Microscope (UKB Microscope Core Facility). Total areas were imaged with a 20x objective utilizing 488, 555 and 633 filters.

Axonal density quantification

Analysis in mouse mPFC sections

Images of the mPFC were matched, based on DAPI signal, with corresponding levels from coronal adult mouse Allen Brain Reference Atlas (http://atlas.brain-map.org) in Fiji (https://imagej.net/software/fiji/). Subsequently, upper (Layers I, II/III) and deeper (Layers V & VI) layers and PFC sub-regions (anterior cingulate (AC), infralimbic (IL) and prelimbic (PL) cortex) were defined and used to define the areas for axonal density quantification. These areas were subjected to Digital Enhancement of Fibers with Noise Elimination (DEFiNE) plugin[3] on Fiji to quantify the DA axonal density. Briefly, autofluorescence signal and non-axonal particles were removed from the z-stack images. Afterwards, an axonal fiber quantification step was performed on maximum intensity projection (MIP) images by quantifying only pixel intensities above measured threshold. Axonal occupation index was defined as the percentage of the area covered by axonal fibers: the quantified axonal output area (μm^2) divided by the respective total area (μm^2) *100.

Analysis in human FCD type 2b specimen

For each human FCD type 2b specimen, control and FCD type 2b areas were pre-defined based on H&E staining and distribution of NeuN-positive neurons. The control area was defined as an area with proper cortical lamination and absence of any cytological abnormalities, the FCD type 2b area was defined based on its disrupted cortical lamination and cytological abnormal cells. A seminal study using a combination of stereological EEG and histology found that 'the epileptogenic zone corresponded to histologically defined FCD in 82% of the cases'.[4] Thus, we

have here used neuroanatomically regular cortex as control tissue that had to be neurosurgically removed to obtain access to the dysplastic lesion. Control and FCD type 2b areas were outlined as contours in Neurolucida software (MBF Bioscience). Cortical layer markers (see Processing of human tissue) in combination with the global neuronal marker NeuN facilitated cortical layer classification into upper (Layer I & II), middle (Layer III & IV) and deeper (Layer V & VI) layers. After defining these compartments in the control area, the outlines were overlaid on the FCD type 2b area and "layers" were outlined accordingly. Afterwards, TH-labelled DA axons were manually traced throughout the serial sections only within the defined contours in every section. During tracings, the axonal diameter was changed based on axon thickness. Axonal varicosities, defined as swellings with a diameter of more than 0.2 μm, were marked. Lastly, a 3D reconstruction of the total serial section tracings was created for every specimen and was exported to Neurolucida Explorer (MBF Bioscience) for final data acquisition. The occupation index was calculated as percentage of total axonal length for each analyzed area normalized for the total area*100. Number of varicosities were normalized for the total axonal length in each respective area to define varicosity density.

Drd1/DRD1 and *Drd2/DRD2* receptor mRNA quantification

Drd1/DRD1 and *Drd2/DRD2* mRNA expression was assessed by measuring the quantity of fluorescent puncta (each puncta representing a single mRNA transcript) within each cell soma. For the analysis of receptor expression in mouse mPFC and human FCD type 2b sections, areas were defined in the same manner as described above for the axonal density quantifications. Several regions of interest (ROIs) selected within these areas were then used in 2D to train the Cellpose machine learning tool for cell segmentation.[5,6] The trained models for each marker (GFP, RFP and NeuN for mouse; SMI32 and NeuN for human) were utilized to segment cells for mouse in 3D *via* the Cellpose-napari plugin (https://cellpose-napari.readthedocs.io/en/latest/) using a stitch threshold of 0.3 or for human in 2D *via* the Cellpose GUI (v2.0). Neurons with minimal or indistinguishable GFP/RFP signals were excluded from the analysis because they could not be clearly assigned to one of the groups. Then, *Drd1/DRD1* and *Drd2/DRD2* puncta quantification was performed with Radial Symmetry-FISH (RS-FISH) plugin.[7] For quantifications, RANSAC model fitting was used and the sigma and threshold were defined as 0.5 and 0.00595 for images

from mouse mPFC and as 1.5 and 0.00551 for images of human specimen. *Drd1/DRD1 and Drd2/DRD2* puncta were only quantified within segmented NeuN cell somas. Final quantification of puncta per cell was achieved by using the “mask filtering option” in the RS-FISH plugin. Segmented masks of GFP/RFP cells in mouse mPFC were used to identify electroporated neurons; segmented masks of SMI32 cells in human specimen were used to identify dysmorphic neurons. This was achieved by finding the masks corresponding to the GFP, RFP and SMI32 positive neurons from the negative ones using the median intensity measurement 2D/3D function in MorphoLibJ plugin[8] in Fiji.

Phospho-S6 expression analysis

To confirm the overactivation of the mTOR-signaling pathway in the transfected neurons of the WT-mTOR and p-Leu2427Pro mPFC, we stained for Phospho-S6 (Ser240/244), a marker of mTORC1 activation, as described above (Immunohistochemistry - Mouse tissue). The overall mPFC area was imaged with a 20x air objective with the Visitron VisiScope Confocal Spinning Disk Microscope (UKB Microscope Core Facility). For the quantification, the overall mPFC area was included. RFP+/GFP+ neurons were identified through thresholding in Fiji, followed by the creation of binary masks. Watershed segmentation was then applied to improve separation between neighboring cells, and the "analyze particles" function was used to segment individual transfected neurons in the entire mPFC region. For RFP+ and GFP+ neurons, thresholds were set at 80-infinity and 150-infinity, respectively, to ensure accurate segmentation. These segmented particles were then combined using the OR function, and the resulting selection was overlaid on the pS6 channel. Areas outside the selection were cleared, isolating pS6 expression exclusively in the RFP+/GFP+ neurons. This process was repeated, with the OR function applied again and clearing the area within the selections to capture pS6 expression only in the surrounding RFP-/GFP- neurons. The raw mean grey values were measured within these separate selections and plotted.

TH-NET colocalization analysis

To analyze whether a subset of the TH-expressing processes in the mPFC are NA axons, we stained for TH and sodium-dependent noradrenaline transporter (NET/SLC6A2) as described in Material

and Methods (Immunohistochemistry - Mouse tissue). Area imaging and distinctions of upper and deeper layer areas in the mPFC was performed as described for DA axonal analysis. For the quantification, TH channel was thresholded in Fiji and converted to a binary image and then the area occupied by the axons was measured. Afterwards, this thresholded area was selected and overlaid on the NET channel and the non-selected area was cleared out, to obtain only the colocalizing area with double labelled axons. Then, the percent colocalization area was measured by dividing the colocalized area by the total area occupied by TH axons*100.

Statistical analysis

Statistical tests and visualization graphs were made in R Studio and GraphPad Prism version 10.4.1.[9] First, data distribution for each data set was graphically visualized with histograms and Q-Q plots and tested for normal distribution with the Shapiro-Wilk test. If the normality test was passed the following tests were performed: paired or unpaired t-tests (where appropriate p-values were adjusted for multiple comparisons using the Bonferroni-Dunn method), one-way ANOVA with repeated measures followed by Tukey correction for multiple comparisons between groups or two-way ANOVA with repeated measures followed by Bonferroni correction for multiple comparisons were applied. If the normality test was not passed, non-parametric Mann-Whitney-Wilcoxon test or Kruskal-Wallis test followed by post-hoc Dunn's test with Bonferroni correction test for multiple comparisons was applied. The statistical tests applied are indicated in the figure legends. Boxplots and violin plots were utilized to show the value distribution, where additionally mean values are shown with data points and median values are indicated by horizonal lines within the boxplots. Additionally, *Drd1/DRD1* and *Drd2/DRD2* mRNA transcript expression values are reported in the text as mean values ±SEM. Correlation analysis was performed with Spearman test method and correlation coefficient and P-values are reported in the figures. $^{*}P < .05$, $^{**}P < .01$, $^{***}P < .001$, $^{****}P < .0001$.

1. Najm I, Lal D, Alonso Vanegas M, et al. The ILAE consensus classification of focal cortical dysplasia: An update proposed by an ad hoc task force of the ILAE diagnostic methods commission. *Epilepsia*. 2022;63(8):1899-1919.
2. Nakagawa JM, Donkels C, Fauser S, et al. Characterization of focal cortical dysplasia with balloon cells by layer-specific markers: Evidence for differential vulnerability of interneurons. *Epilepsia*. 2017;58(4):635-645.
3. Powell JM, Plummer NW, Scappini EL, Tucker CJ, Jensen P. DEFiNE: A method for enhancement and quantification of fluorescently labeled axons. *Front Neuroanat*. 2019;12(January):1-11.
4. Chassoux F, Devaux B, Landré E, et al. Stereoelectroencephalography in focal cortical dysplasia: A 3D approach to delineating the dysplastic cortex. *Brain*. 2000;123(8):1733-1751.
5. Stringer C, Wang T, Michaelos M, Pachitariu M. Cellpose: a generalist algorithm for cellular segmentation. *Nat Methods*. 2021;18(1):100-106.
6. Pachitariu M, Stringer C. Cellpose 2.0: how to train your own model. *Nat Methods*. 2022;19(12):1634-1641.
7. Bahry E, Breimann L, Zouinkhi M, et al. RS-FISH: precise, interactive, fast, and scalable FISH spot detection. *Nat Methods*. 2022;19(12):1563-1567.
8. Legland D, Arganda-Carreras I, Andrey P. MorphoLibJ: Integrated library and plugins for mathematical morphology with ImageJ. *Bioinformatics*. 2016;32(22):3532-3534.
9. Team RC. R: A Language and Environment for Statistical Computing. Published online 2020. https://www.r-project.org/
